# Fluoride export (FEX) proteins from fungi, plants and animals are 'single barreled' channels containing one functional and one vestigial ion pore

**DOI:** 10.1371/journal.pone.0177096

**Published:** 2017-05-04

**Authors:** Tetyana Berbasova, Sunitha Nallur, Taylor Sells, Kathryn D. Smith, Patricia B. Gordon, Susan Lori Tausta, Scott A. Strobel

**Affiliations:** 1 Department of Molecular Biophysics and Biochemistry, Yale University, New Haven, Connecticut, United States of America; 2 Chemical Biology Institute, Yale University, West Haven, Connecticut, United States of America; 3 Division of Basic Sciences, Fred Hutchinson Cancer Research Center, Seattle, Washington, United States of America; University of South Florida, UNITED STATES

## Abstract

The fluoride export protein (FEX) in yeast and other fungi provides tolerance to fluoride (F^-^), an environmentally ubiquitous anion. FEX efficiently eliminates intracellular fluoride that otherwise would accumulate at toxic concentrations. The FEX homolog in bacteria, Fluc, is a ‘double-barreled’ channel formed by dimerization of two identical or similar subunits. FEX in yeast and other eukaryotes is a monomer resulting from covalent fusion of the two subunits. As a result, both potential fluoride pores are created from different parts of the same protein. Here we identify FEX proteins from two multicellular eukaryotes, a plant *Arabidopsis thaliana* and an animal *Amphimedon queenslandica*, by demonstrating significant fluoride tolerance when these proteins are heterologously expressed in the yeast *Saccharomyces cerevisiae*. Residues important for eukaryotic FEX function were determined by phylogenetic sequence alignment and functional analysis using a yeast growth assay. Key residues of the fluoride channel are conserved in only one of the two potential fluoride-transporting pores. FEX activity is abolished upon mutation of residues in this conserved pore, suggesting that only one of the pores is functional. The same topology is conserved for the newly identified FEX proteins from plant and animal. These data suggest that FEX family of fluoride channels in eukaryotes are ‘single-barreled’ transporters containing one functional pore and a second non-functional vestigial remnant of a homologous gene fusion event.

## Introduction

Fluoride is a toxin that is widespread in nature. The average fluoride concentration in the ocean is 70 μM [1], but much higher fluoride levels (>100 mM) can be detected near active volcanoes [2–4]. Fluoridation, industrial pollution and dental product waste also contribute to distribution of this anion in the environment [1]. Harmful effects of high fluoride concentrations on human health have been documented, but its beneficial impact at low concentration is broadly recognized, leading to its addition to most dental products and municipal drinking water [5]. The World Health Organization recommends 1.5 mg/L (79 μM) of fluoride ion in drinking water [6].

The impact of fluoride on the environment and human health are based on its chemical properties. Fluoride is a non-nucleophilic hydroxide mimic because of its small atomic radius and high basicity [7]. Furthermore, AlF_4_^-^, a complex that is readily formed in the presence of aluminum ions, is an analog of phosphate ion that can serve as a transition state mimic to inhibit phosphoryl transfer reactions [8]. Therefore, fluoride ions may inhibit enzymes that rely on hydroxyl-forming intermediates or reactive phosphate, essentially halting most proliferative processes [9]. This inhibition causes the termination of crucial biological events including glycolysis, phosphorylation, nucleotide synthesis and polymerization [5, 7, 9].

The ubiquity of fluoride in nature has contributed to the evolutionary development of mechanisms to control intracellular F^-^ levels in microorganisms. The recent discovery of a fluoride riboswitch in eubacteria and archaea provided the first clues about the cellular mechanisms for circumventing fluoride toxicity [10]. Breaker and peers identified a riboswitch controlling expression of many genes, including two transmembrane proteins of unknown function. These two distinct families of membrane proteins, CLC^F^ and crcB, facilitate fluoride export across cell membranes. CLC^F^ is a F^-^/H^+^ transporter that belongs to the CLC superfamily [11]. In contrast, crcB is a unique and broadly distributed family of proteins [12]. Further characterization of bacterial crcB (later renamed Fluc) showed that these proteins are ion channels with high selectivity (>10,000 fold) for fluoride over chloride [12].

**Fig 1:**
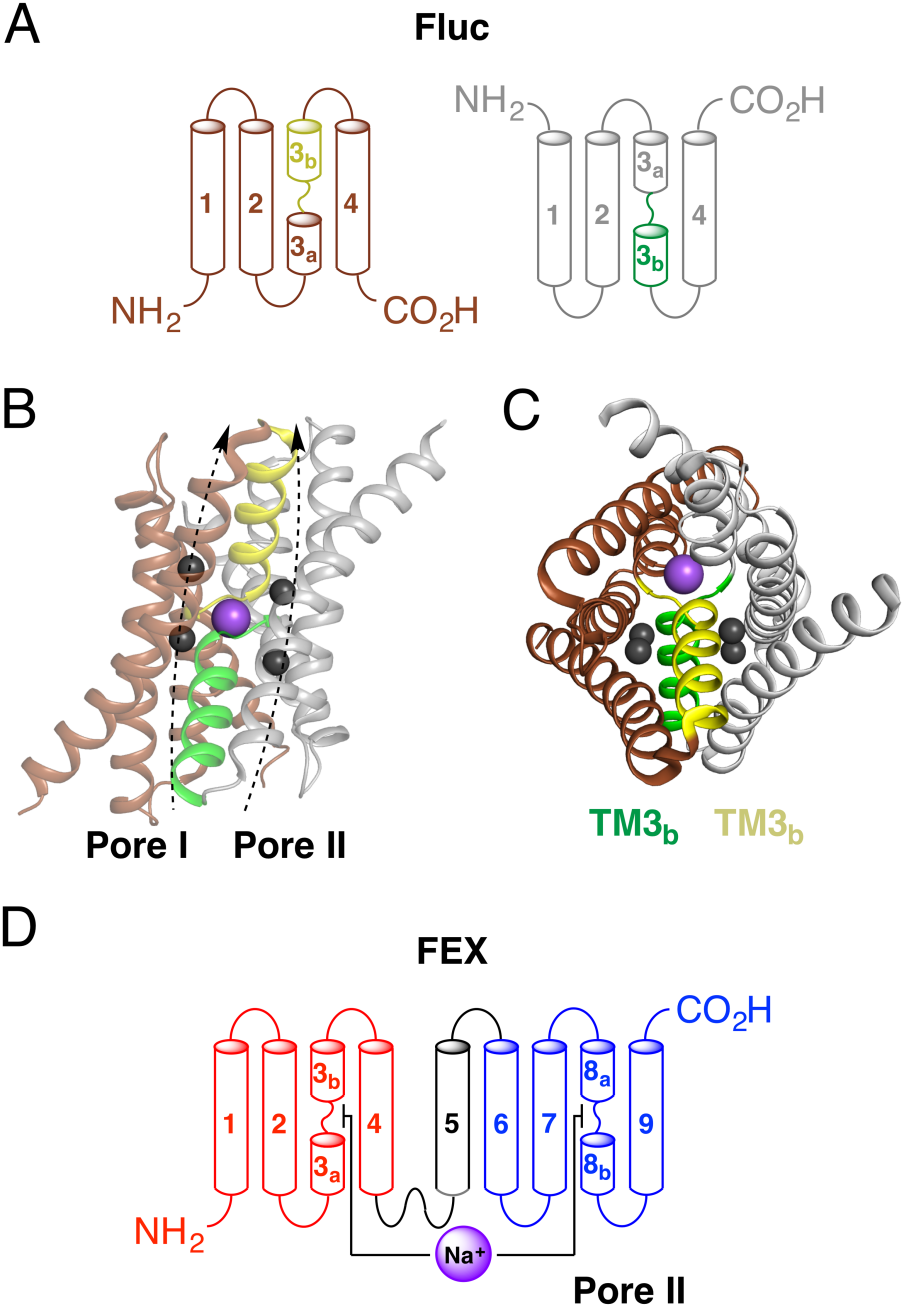
Structural arrangement of fluoride export proteins. (A) Topology model of bacterial Fluc protein. (B) Tertiary structure of Fluc-*Bp* (PDB ID: 5FXB) showing two pores with fluoride (dark grey) ions. Sodium ion (purple) is in the middle of the protein dimer. Two monomers are colored brown and light grey. (C) Arrangement of transmembrane helices (top view). Helices TM3b from different monomers (yellow and green) separate two pores and have residues belonging to two different pores but located on the same helix. (D) Topology model of eukaryotic FEX protein with proposed interactions between TM3 and TM8 based on Fluc crystal structure.

Fluc is an antiparallel dimer that forms two active fluoride pores [13]. Each monomer has four transmembrane α-helices (Fig 1A) [12]. In different organisms the functional unit may be a homodimer or a heterodimer, but the transmembrane topology is highly conserved. Recently, Stockbridge *et al*. reported the crystal structures of the bacterial exporter Fluc from *E*. *coli* and *B*. *pertussis* [14]. The structures reveal a double-barreled two-pore assembly of fluoride-specific ion channels (Fig 1B). The crystal structures also showed that transmembrane helix 3 (TM3) is broken into two halves, TM3a and TM3b. The two TM3 half helices from separate monomers physically separate the two pores (Fig 1C), with residues on two sides of the same helix occupying positions in each pore. This arrangement is achieved through the formation of an antiparallel dimer because two TM3b helices are essential for the dual pore architecture. Two TM3 helices from separate monomers cross at the center of the membrane and form a putative sodium binding site, which suggests the cation is structurally important.

Fluc is proposed to use aromatic and hydrogen bond donating amino acids within its pores to transport fluoride ions. There are highly conserved phenylalanines located in TM3 that face into the pore and adopt a side-to-face ‘box’-like arrangement for interaction with fluoride [14]. In addition, residues facing the pore in TM4 are different hydrogen bond donors of low sequence identity. Every fourth residue in TM4 varies between N, S, T, H or Y, which is proposed to provide a polar track needed for recognition of fluoride ions [14, 15].

A two-pore model of Fluc was confirmed by mutagenesis of the four highly conserved phenylalanine residues that are located in the pore [14, 15]. In the homodimeric Fluc from *E*. *coli*, F80 and F83 are both located in TM3b, but F80 is in one pore and F83 is in another pore (one F80 and one F83 in Pore I and one F80 and one F83 in Pore II) [15]. Because of this, each Fluc monomer contributes a phenylalanine to each pore. Mutations of these two phenylalanines in the monomer (four mutations within the homodimer) made Fluc inactive and the fluoride ions were absent in the crystal structure of these mutants. Last *et al*. also tested constructs containing two fused monomers [15]. This protein does not form a homodimer to achieve function, but is fully funtional as a monomer with two domains making it possible to test mutations in one pore, while not affecting the second. Mutation of the two Phe to Ile in one pore inactivated that pore but the other pore remained functional in this concatameric Fluc construct.

Fluoride channels are also conserved in eukaryotes and are termed FEX, for Fluoride EXport protein [16]. Previous studies conducted in three fungal organisms, *S*. *cerevisiae*, *C*. *albicans* and *N*. *crassa*, revealed that all three rely on *FEX* to bypass fluoride toxicity and avoid F^-^ accumulation inside cells. FEX proteins have also been identified and used in *S*. *pombe* as a non-auxotrophic selectable marker that is compatible with CRISPR [17].

*S*. *cerevisiae* contains two genes that encode FEX proteins. These two proteins differ from each other in only three amino acids, suggesting that the two copies are the result of recent gene duplication. Single deletion of either gene has no effect on yeast sensitivity to fluoride [16]. However, deletion of both *FEX* genes generates a fluoride sensitive strain with an IC_50_ that is approximately 1000-fold lower than the wild-type strain. Both proteins, Fex1p-*Sc* and Fex2p-*Sc*, are constitutively expressed in nearly equal amounts in the yeast plasma membrane [18]. Each protein consists of two homologous domains joined into a single protein by a transmembrane linker (Fig 1D) that forces an antiparallel orientation of the N-terminal and C-terminal domains [18]. This domain arrangement is analogous to the engineered fusion constructs that were created for the study of Fluc [15].

Sequence alignment of the prokaryotic proteins and the N and C terminal domains of the eukaryotic protein identified conserved motifs that occur in both domains [18]. There is a G(A)xxxR sequence in the first helix and an N in the middle of the second helix of each FEX domain or Fluc monomer. Various mutations of these conserved residues within FEX suggested that the residues in the C-terminal domain have greater effects on yeast fluoride tolerance than the equivalent residues in the N-terminal domain, though the cause of this asymmetric effect on protein activity was not clear.

The crystal structures of Fluc, combined with our earlier mutagenesis study, inspired the investigation of the asymmetrical nature of the two pores in the yeast FEX protein. Herein we report the identification of residues essential for fluoride export using sequence alignment, structural modeling, mutagenesis studies, and yeast growth tolerance in fluoride. The eukaryotic fluoride channel is rendered inactive by mutation(s) located in Pore II but equivalent mutations in Pore I have little or no effect. We also demonstrate that homologous proteins in higher eukaryotes can rescue the fluoride sensitive phenotype and have the same asymmetric sequence conservation in Pore II. These results suggest that among eukaryotic fluoride channels, Pore II is functional for fluoride export while Pore I has lost its ancestral function through evolutionary drift, resulting in a vestigial pore.

## Results

### Identification of conserved motifs

**Fig 2:**
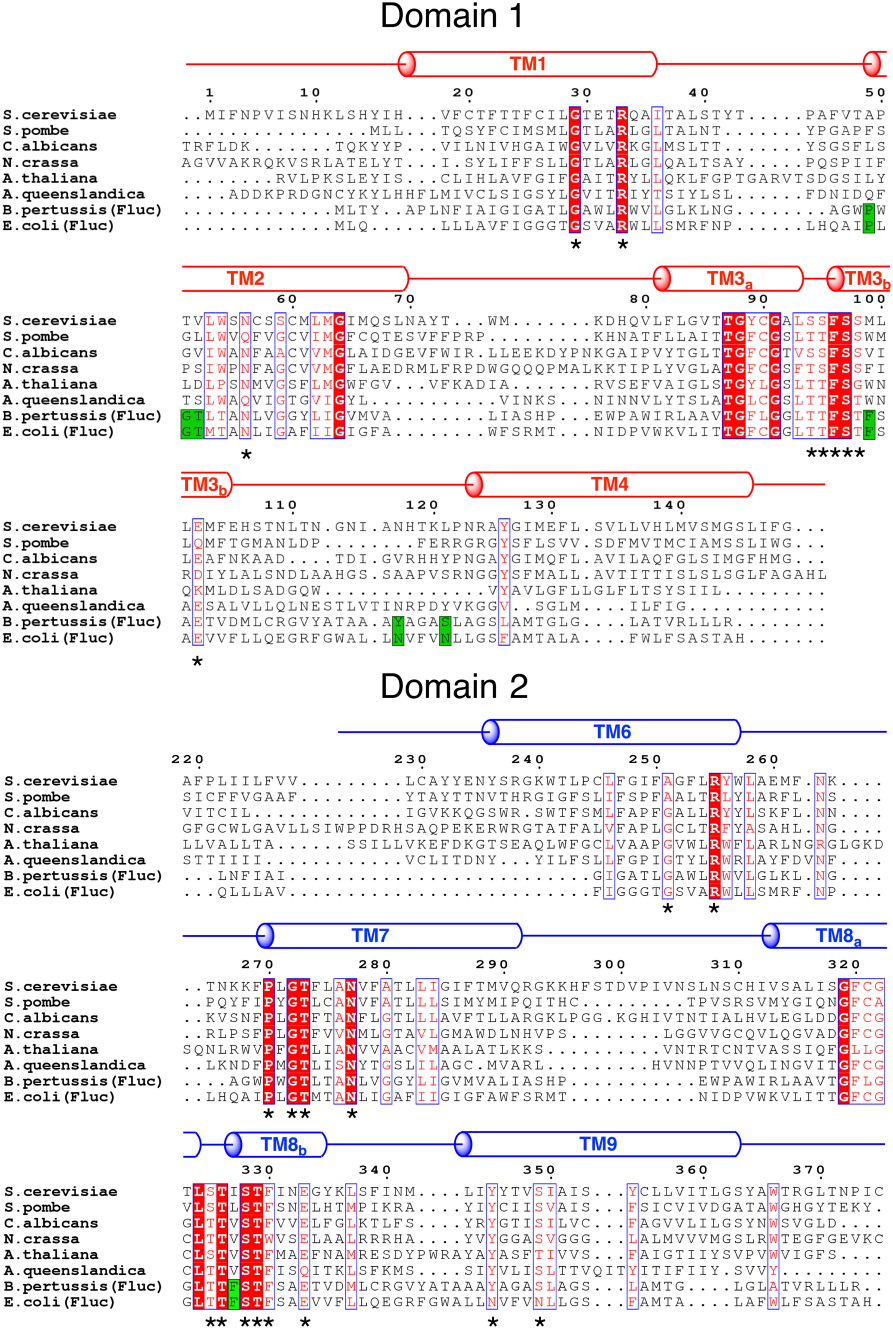
Alignment of protein sequences from six eukaryotic and two bacterial fluoride channels. Transmembrane regions were predicted using TMHMM and TMpred servers. Conserved residues for all eukaryotic FEX proteins are highlighted with asterisks. Amino acid numbering corresponds to the protein sequence of Fex1p from *S*. *cerevisiae*. The residues highlighted in green are present within both pores of Fluc.

The sequence alignment of eukaryotic FEX proteins can provide clues about conservation and function. Because the sequence similarity in the FEX protein family is low, residues that are conserved in all proteins are expected to be important for pore formation. We aligned 190 FEX-like proteins from various eukaryotic organisms. Alignment of FEX with the pore-facing residues identified in Fluc suggests which residues create the pores in the eukaryotic protein (Fig 2). There is clear alignment for the key residues in Pore II but a striking lack of equivalent residues in Pore I. Four motifs are conserved in all FEX proteins: ooFooX, ooXooF, PxGTxxxN, and YxxxS(T) (X—any amino acid, o—serine or threonine). These motifs are in the hydrophobic helices TM3, TM8, TM7 and TM9, respectively.

Helices TM7, TM8, and TM9 are more similar to Fluc helices TM2, TM3 and TM4 than the equivalent helices TM2, TM3, and TM4 in FEX. For example, a motif with high sequence conservation, PxGTxxxN, is found in TM7 but is not present within the analogous segment of TM2. In TM3 and TM8, only half of the four F residues are conserved in FEX. Helix TM3 has a F in the beginning of TM3b and TM8 has a F (and rarely W) in the third amino acid of TM8b. Both of these residues overlap with F residues that face Pore II in Fluc, but both of the F residues in Pore I are absent. In Fex1p-*Sc* they are I and M and these residues are not conserved among other eukaryotes. There is also a well conserved Glu at the end of TM8. There is much less sequence conservation at the equivalent residue in TM3. A motif of strong similarity, YxxxS(T), is identified in TM9 of FEX, but not in its sister helix, TM4. This fragment overlaps with the polar track residues of Pore II in Fluc.

Although the residues within the pores are not conserved in both domains of FEX, other residues in TM3 and TM8 do show a symmetrical pattern of sequence conservation. These residues are near the sodium binding site and some of them coordinate a putative sodium ion using four backbone carbonyl groups [14]. The general sequence is ooF_II_ooF_II_, where *o* is either S or T, and *F*_*II*_ is F in Pore II but is not a conserved residue in Pore I (see above). In Fluc, the first set of consecutive S/T (ooF_II_ooF_II_) forms a TM3 break where two TM3 helices from the separate monomers intersect (Fig 1C) [14]. The second Ser/Thr pair (ooF_II_ooF_II_) initiates the helical turn of TM3b and space out two residues that face different pores. Sequence alignment showed that the hydroxyl-containing amino acids of the ooF_II_ooF_II_ fragment are in both domains of FEX, suggesting their importance for protein function even though equivalent residues in Fluc are not part of the pore.

**Fig 3:**
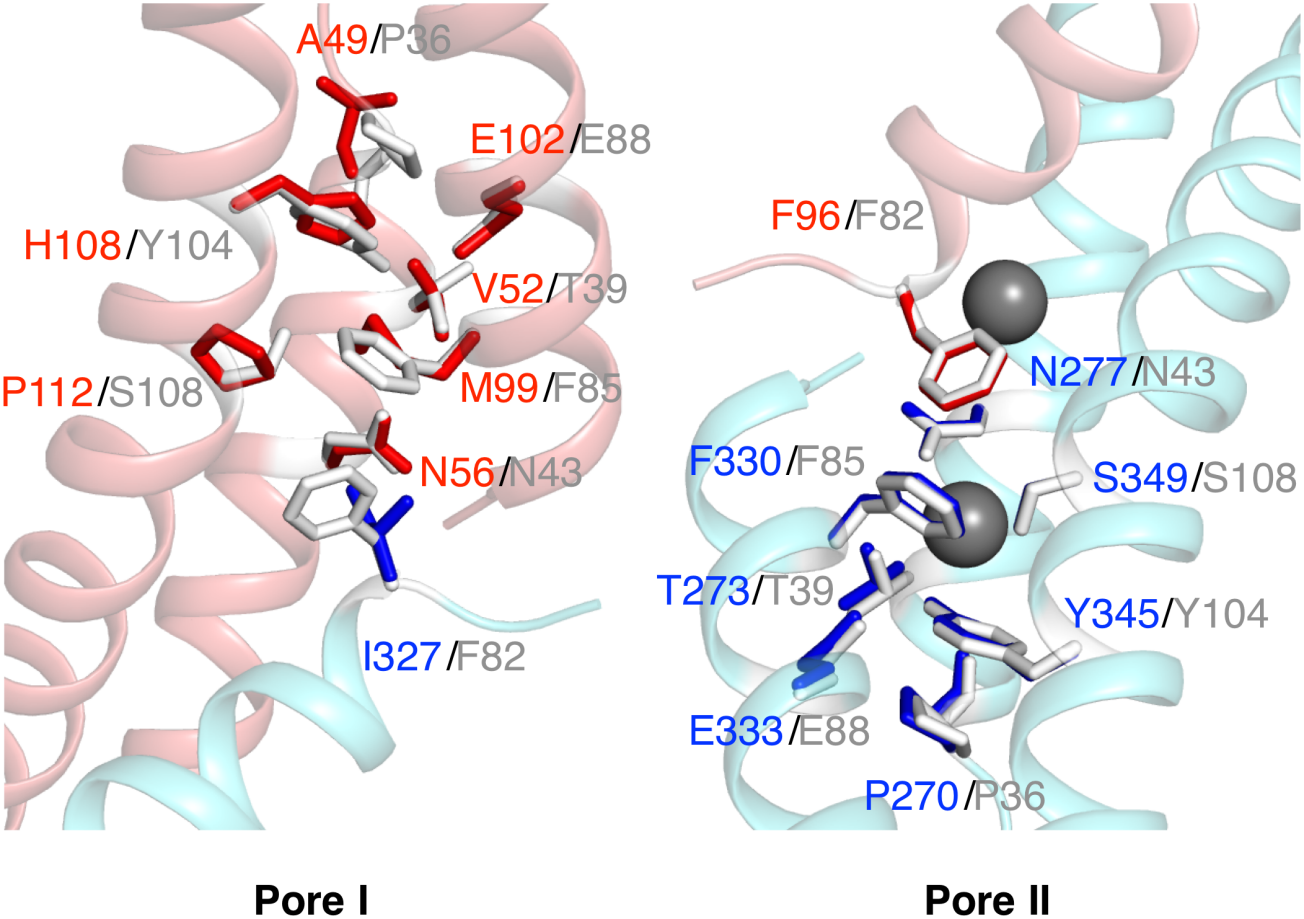
Model of Fex1p from *S*. *cerevisiae*. Comparison of the residues in two pores for Fex1p-*Sc* (red residues are in N-terminal domain and blue are in C-terminal domain) and Fluc-*Bp* (white). Residues in Pore II and not Pore I of FEX are similar to bacterial Fluc.

We built a homology model of FEX based upon the homodimer structure of Fluc, specifically the crystal stucture of Fluc from *B*. *pertussis* (denoted Fluc-*Bp*), to serve as a framework for designing mutagenesis studies of FEX. Homology modeling output for the N-terminal and C-terminal domains of Fex1p-*Sc* showed sequence identity to Fluc-*Bp* of 19% and 23%, respectively. The models of the two domains were combined and overlaid with the Fluc-*Bp* crystal structure to estimate the location and compare the residues in the two pores (Fig 3). This model further demonstrates that Pore I is highly divergent from the bacterial ortholog while Pore II has most of the key residues.

In this homology model, Pore I consists of helices TM1, TM2, TM4 and residues facing Pore I from the helices TM3a and TM8b. Pore I of Fex1p-*Sc* and Fluc-*Bp* appear to have only a few residues in common (Fig 3, left side), including N56 in the middle of Pore I that overlaps with N43 of Fluc. There is also E102 near the extracellular loop that is identical to E88 of the bacterial ortholog. The rest of the pore-lining residues have no similarity between the two proteins, including the two critical Phes in TM3b and TM8b. Furthermore, there are no Phes at nearby residues that could substitute for the nonpolar side chains of M99 and I327. T39 of Fluc is replaced by V52 in TM2 of FEX, which breaks the conserved PxGTxxxN sequence. Finally, the TM4 helix of FEX does not contain the polar track of residues of the bacterial TM4, leading to a loss of the YxxxS(T) motif. The TM4 sequence alignment is so divergent between FEX proteins and Fluc that the model cannot accurately predict the helix. This leads to a vague estimation of residues in Pore I from TM4, but the sequence of this helix does not include properly spaced H-donating residues that could serve as the polar track (Fig 2).

In contrast, Pore II retains many of the conserved amino acids that are present in the bacterial protein (Fig 3, right side). Pore II of FEX is comprised of helices TM6, TM7, TM9 and residues facing Pore II from helices TM3a and TM8b. Bacterial residues F85 and F82 are equivalent to F96 and F330 of FEX. A hydrogen bond donor, T39 in Fluc, is present as T273 in FEX (the PxGTxxxN motif), and at least part of the polar track residues, Y345 and S349, are observed in TM9 (the YxxxS motif).

In contrast to the asymmetric conservation of residues in Pore I and Pore II, the predicted sodium binding site includes the conserved ooF_II_ooF_II_ motif with conserved S/T located in both the N- and C-terminal domains of FEX. In the structure of Fluc, the sodium ion is coordinated through the main chain carbonyl of these S and T residues while their side chains make H-bonding interactions with each other. Although there are conserved residues in the N-terminal domain as part of this ooF_II_ooF_II_ motif, the conserved residues are not in the pore. Instead of directly contacting fluoride, they are likely to help form a cation binding site analogous to equivalent residues in Fluc.

### Mutations in Pore II show complete loss of protein function

**Fig 4:**
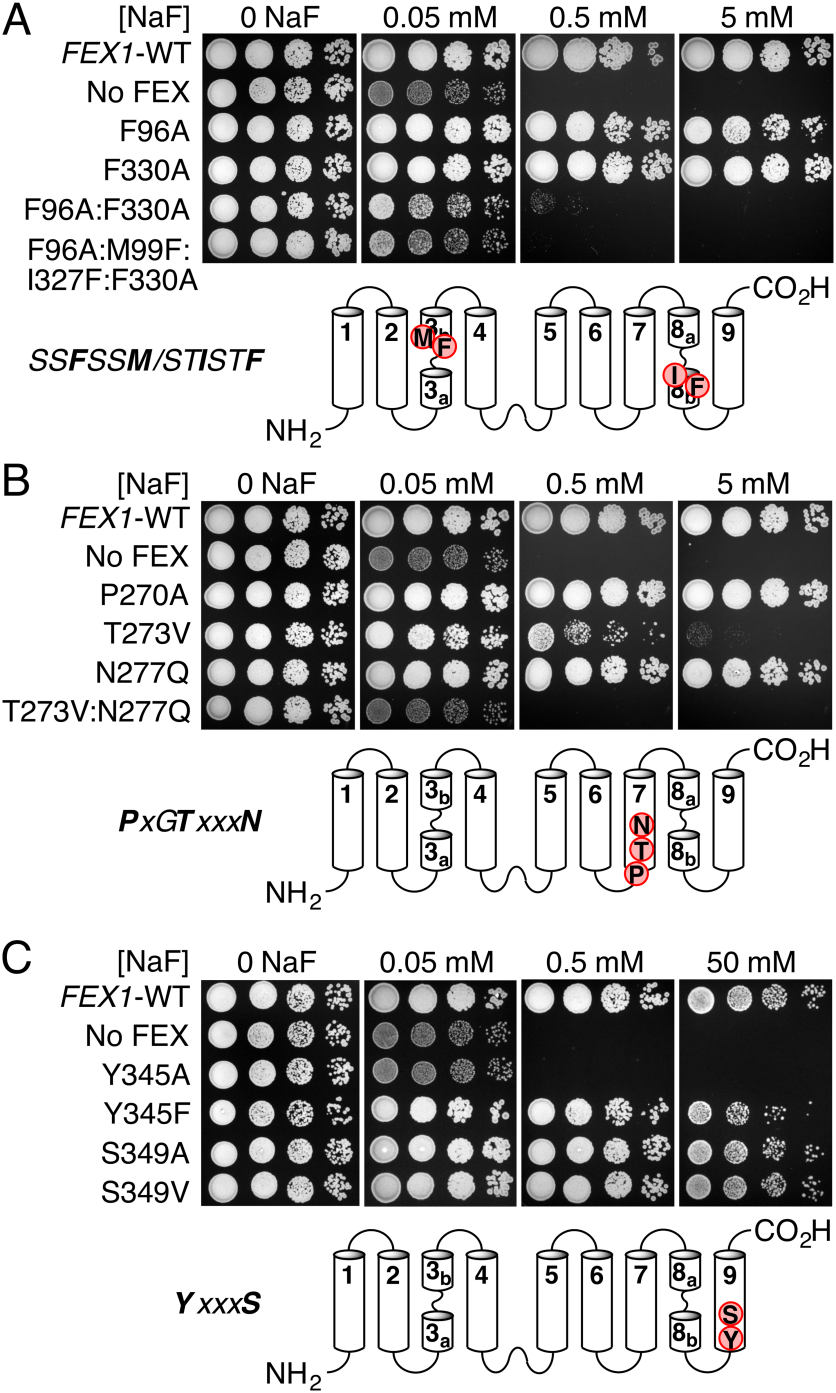
Functional analysis of conserved fragments in FEX. *A*, mutations to pore-lining Phe located in TM3 and TM8. *B*, mutations to PxGTxxxN motif located in TM7. *C*, mutations to YxxxS fragment located in TM9. The *fex1Δfex2Δ* strain was transformed with wild-type pRS416-*FEX1* (WT), empty vector pRS416 (No FEX) or Fex1p mutants in pRS416-*FEX1*. Serial dilutions of yeast cultures expressing the indicated mutants were incubated on YPD media with different concentrations of NaF.

We functionally evaluated conserved residues within the FEX protein by testing for the ability of FEX mutants to rescue the growth of fluoride-sensitive yeast. FEX mutations were introduced to a rescue plasmid, pRS416-*FEX1*, and compared to the original plasmid (denoted *FEX1*-WT) and the empty vector (pRS416 denoted ‘no FEX’). Plasmids were transformed into a fluoride sensitive yeast strain (*fex1Δfex2Δ*) and the yeast were scored for their growth phenotypes at different concentrations of fluoride (Fig 4). Growth tolerance curves were used to get a quantitative estimate of FEX function by measuring IC_50_ values (Table 1) and comparing the ratio of mutant and WT values (Fig 4).

**Table 1:** Effect of mutations to residues in the hypothetical pores of Fex1p.

Conserved motif	Fex1p mutant	IC_50_ (mM)	Fold-change	TM helix	Location
	WT	49 ± 13	1.0		
	Empty vector	0.066 ± 0.004	740		
PxGT*xxx*N	P270A	47 ± 1	1.1	TM7	Pore II
	T273V	0.41 ± 0.14	120	TM7	Pore II
	N277Q	18 ± 5	2.7	TM7	Pore II
	T273V:N277Q	0.045 ± 0.003	>1000	TM7	Pore II
ooFooM*xx*E/ooIooF*xx*E	F96A	4.3 ± 3.2	11	TM3b	Pore II
	F330A	34 ± 20	1.4	TM8b	Pore II
	F96A:F330A	0.040 ± 0.009	>1000	TM3b/TM8b	Pore II (Phe removed)
	F96A:M99F: I327F:F330A	0.043 ± 0.015	>1000	TM3b/TM8b	Pore I (Phe added)/Pore II (Phe removed)
	E102A	52 ± 5	0.9	TM3b	Pore I
	E333A	0.56 ± 0.20	88	TM8b	Pore II
	E333Q	23 ± 3	2.1	TM8b	Pore II
	E333K	0.069 ± 0.002	710	TM8b	Pore II
	E102A:E333A	0.041 ± 0.008	>1000	TM3b/TM8b	Pore I/Pore II
Y*xxx*S(T)	Y345F	29 ± 8	1.7	TM9	Pore II
	Y345A	0.054 ± 0.003	910	TM9	Pore II
	S349A	43 ± 9	1.2	TM9	Pore II
	S349V	47 ± 7	1.0	TM9	Pore II

Next, we examined the conserved phenylalanines in Pore II (F96 and F330) and corresponding residues in Pore I (M99 and I327) that together would form the phenylalanine ‘box’-like fragment (Fig 2). Residue F96 is in helix TM3 of the N-terminal domain, and forms part of Pore II (Fig 3). The single mutation F96A causes a 11-fold loss of yeast growth tolerance to fluoride (Fig 4A, Table 1). Residue F330 in TM8 of the C-terminal domain faces Pore II and equivalent residue has aromatic side chain in other FEX proteins (S1 Fig). The single substitution F330A in Pore II had a modest influence on FEX activity with only a 1.4-fold loss in the IC_50_ value (Table 1). However, the combination of both mutations (F96A:F330A) resulted in an IC_50_ in the range of the yeast strain without FEX (>1000-fold loss). We confirmed expression of FEX proteins using Western blotting with HA-tagged version of Fex1p in the equivalent plasmid (S2 Fig). This demonstrates that mutations of F in Pore II result in complete FEX inactivation.

There are conserved E residues, E102 and E333, located symmetrically in each pore three amino acids downstream of M99 and F330 in TM3 and TM8, respectively. The structural model predicts that E333 is at the opening of Pore II on the cytoplasmic side, while E102 is on the exterior of the membrane in Pore I. We mutated E to A in both pores. Mutation in Pore II but not Pore I had a high impact on FEX activity: E102A and E333A caused a 0.9- and 88-fold decrease in IC_50_ values, respectively (Table 1). As an alternative to E, some FEX proteins have either Q or D in Pore II, and much higher diversity in Pore I, including K in FEX from the plant *A*. *thaliana* (Fig 2). This might be an indication of even further pore degradation in higher eukaryotes. Because the E333A mutation caused such a dramatic change in the IC_50_ value, we also measured the growth tolerance of yeast expressing E333Q or E333K mutants. Mutation E333Q showed only a 2-fold loss in the IC_50_, which suggests the possibility for hydrogen-bonding interactions involving E333 (Table 1). However, change to a positive charge, E333K, made the FEX protein inactive with an IC_50_ in the range of the FEX-deficient yeast (>1000-fold loss). These results suggest the importance of an acidic or proton-donating residue at the entrance of Pore II and is a second example of complete loss of protein function resulting from a mutation in the second pore.

Based on sequence alignment, the motif PxGTxxxN in TM7 (TM2 in Fluc) has almost complete sequence identity among all FEX proteins. To test its importance for fluoride tolerance, we mutated P270, T273, and N277 residues to A, V and Q, respectively, in Fex1p-*Sc* (Fig 4B). In functional viability assays, P270A had very similar fluoride tolerance to *FEX1*-WT (Fig 2A). We substituted T273 with valine because Pore I has a V52 at the position equivalent to T273 (Fig 3). The T273V mutation caused a 120-fold decrease in IC_50_ value. Moreover, when T273V is combined with a previously described mutation N277Q (3-fold loss for the single mutant N277Q) [18], the double mutant T273V:N277Q showed a complete loss (>1000-fold) of fluoride tolerance. The T273V, N277Q double mutant in Pore II is a third example of complete Fex1p-*Sc* inactivation by modifications in the second pore alone.

The motif YxxxS(T) is highly conserved in eukaryotic but not prokaryotic proteins. Mutations of Y345 and S349 in Pore II were investigated in yeast growth assays (Fig 4C). The single mutation Y345A caused >1000-fold loss in the IC_50_ (Table 1). It is one more single point mutation that fully inactivated the FEX protein and it is the fourth example in which mutations in Pore II alone caused complete loss of FEX function. When this Y was mutated instead to another aromatic residue, F, only a 2-fold decrease of growth rescue was observed, suggesting the aromatic character is crucial for function. The minor effect of the Y to F swap supports the hypothesis that the fluoride ions interact not only with hydroxyl groups but also with aromatic side chains [15]. Interestingly, despite the high conservation of S, neither S349A nor S349V mutants impaired yeast growth tolerance.

**Fig 5:**
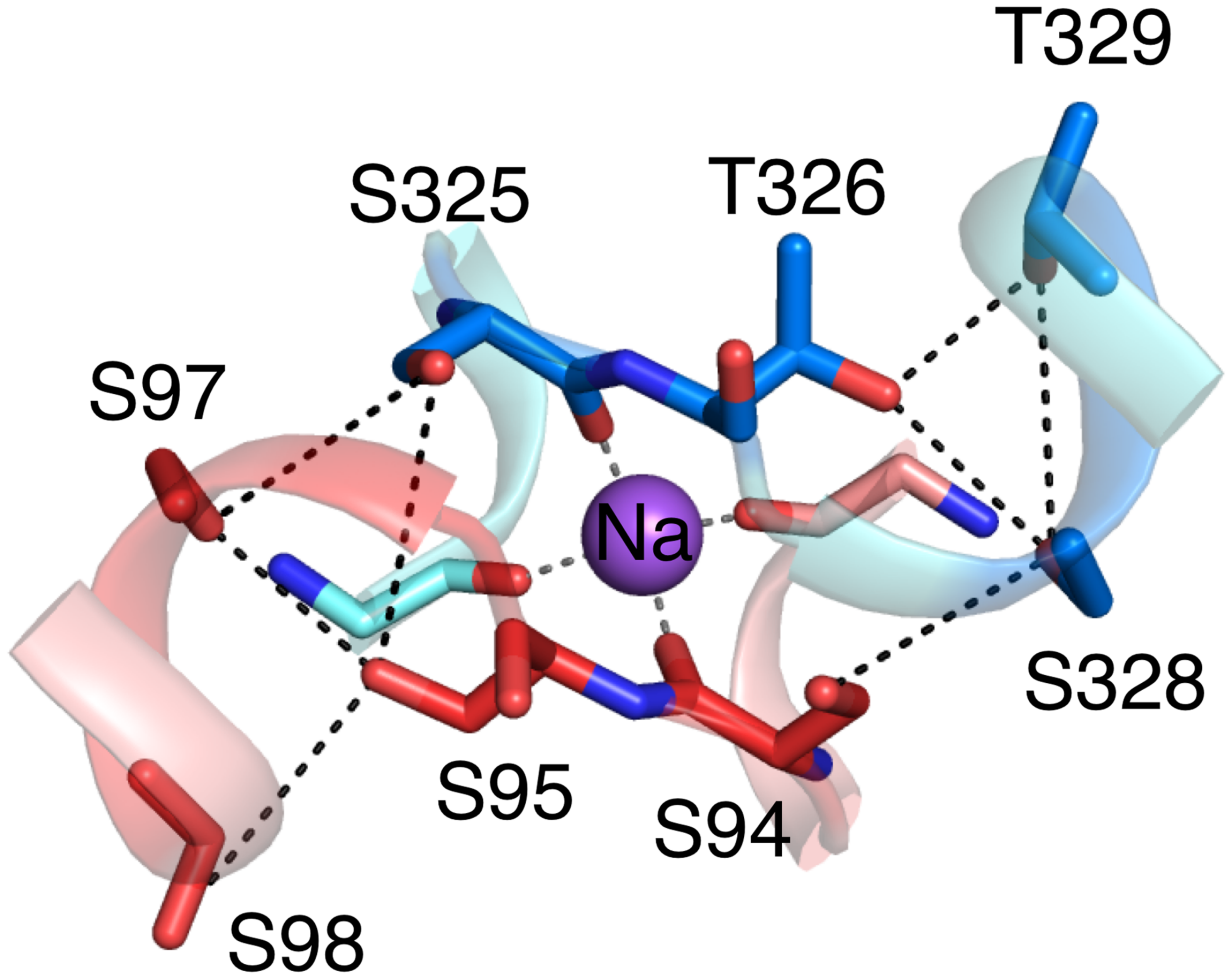
Functional analysis of conserved S/T fragments in domain 1 (red) and domain 2 (blue). Conserved Ser/Thr residues are located near putative Na^+^ binding site.

Despite their location outside of either pore, the S and T residues located in the ooF_II_ooF_II_ motif (TM3 and TM8 in FEX) are highly conserved. The model and sequence alignment of FEX proteins suggests that the S/T-rich motifs could be located near the putative sodium binding site (Fig 5). The first pair of S/T, ooF_II_ooF_II_, connects TM3a and TM3b in the N-terminal domain and TM8a and TM8b in the C-terminal domain, further referred as linker. The second pair of S/T, ooF_II_ooF_II_, is in the first turn helix of TM3b and TM8b. To understand the role of this motif, we mutated residues in symmetric positions in the N-terminal and C-terminal domains of FEX.

Replacement of the serines and threonines ooF_II_ooF_II_ in the linker of either domain (S94V:S95V or S325V:T326V double mutants) resulted in the loss of FEX function (>1000 fold loss) as measured by fluoride tolerance (Table 2). Modification of one soluble linker to hydrophobic residues could perturb not only interactions with the predicted sodium ion but also disrupt interactions with the linker from the other domain. To avoid unfavorable interactions, we created a construct with both linkers mutated to V. The tetra-mutant with V in both domains, S94V:S95V:S325V:T326V, was unable to grow on fluoride, the IC_50_ being >1000-fold less than the rescue plasmid (Table 2). The second S/T motif, ooF_II_ooF_II_, is located at residues S97 and S98 (N-terminal domain) and S328 and T329 (C-terminal domain). In the Fluc structure the side chains of this helix are hydrogen-bonded to the side chains of the linker motif in TM3/TM8 (Fig 5). Mutations to N-terminal domain, S97V:S98V, caused 7.9-fold loss of protein activity, while substitution in C-terminal domain, S328V:T329V, showed a 72-fold loss in the IC_50_ value. The changes in IC_50_ indicate the importance of this fragment but their effects are not as significant as the linker residues in TM3/TM8.

**Table 2:** Effect of mutations at conserved residues in two domains of Fex1p.

Consensus sequences	Fex1p mutant	IC_50_ (mM)	Fold-change	TM helix
	WT	49 ± 13	1.0	
	Empty vector	0.066 ± 0.004	740	
N-terminal domain SSFSSM*xx*E	S94V:S95V	0.043 ± 0.009	>1000	Linker TM3a/b
	S97V:S98V	6.2 ± 4.4	7.9	TM3b
C-terminal domain STISTF*xx*E	S325V:T326V	0.047 ± 0.012	>1000	Linker TM8a/b
	S328V:T329V	0.68 ± 0.15	72	TM8b
N-/C-terminal domains SSFSSM*xx*E/STISTF*xx*E	S94V:S95V: S325V:T326V	0.047 ± 0.003	>1000	Linker TM3a/b Linker TM8a/b

The sequence alignment and mutational data strongly suggest that Pore I is no longer able to transport fluoride ions. Based upon the sequence conservation of Pore II, we attempted to re-engineer Pore I into a functional pore in the background of an inactivated Pore II. We began with a tetra-mutant F96A:M99F:I327F:F330A that introduces two F residues into Pore I and removes them from Pore II. However, the tetra-mutant showed yeast growth in the range of the empty vector (>1000-fold loss) (Fig 4A) suggesting that the presence of the F residues in Pore I is insufficient to restore fluoride export.

The highly conserved PxGT_273_xxxN_277_ motif in Pore II is degraded to APTVLWSN in Pore I where only N is moderately conserved in Pore I of FEX proteins. The magnitude of the T273V mutant in Pore II implies that the presence of V52 of Pore I is one of the residues making Pore I inactive. We edited TM2 to include a full length PxGTxxxN fragment in Pore I in the background of the Phe tetra-mutant by introducing the PxGT portion at residues A49 to V52 to restore the full length PxGTxxxN motif in TM2 (A49P:P50A:T51G:V52T). We retained the spacing between these residues using the three native amino acids from residues 52 to 55. Yeast strains harboring this octa-mutant have a IC_50_ in the range of the strain without FEX (S3 Fig), showing that restoration of these two motifs (F from ooF_II_ooF_II_ and PxGTxxxN) is insufficient to restore activity in Pore I.

Ideally, we would also like to introduce the YxxxS motif into TM4 of Pore I. TM4 is substantially different from TM4 in Fluc or TM9 in FEX, and in general TM4 is highly divergent in eukaryotes (Fig 2). The sequence alignment finds some similarity in TM4 by creating sequence gaps and shifting the YxxxS(T) motif of Fluc to the FEX soluble loop that precedes TM4 (Fig 2). It makes identification of the residue equivalent to Y345 in TM4 difficult. Therefore, we could not rationally determine which residues might be equivalent to the YxxxS motif in Pore I. The fact that such key residues are not conserved is further evidence that Pore I has degraded. As a result, we were unable to identify a set of mutations in Pore I that restored FEX function and conferred fluoride resistance to yeast.

### Identification of functional FEX proteins from other organisms

Managing fluoride toxicity is critical not only for unicellular microorganisms but also for multicellular eukaryotes. Sequence alignment suggests that there are FEX-like proteins in many plants and some animals, however, none of them have been characterized. The yeast functional assay provides a means to quickly test for the ability of a candidate protein to detoxify cells upon fluoride exposure. We chose two genes encoding proteins similar to FEX in higher eukaryotes and determined if their heterologous expression in yeast can provide fluoride tolerance.

**Fig 6:**
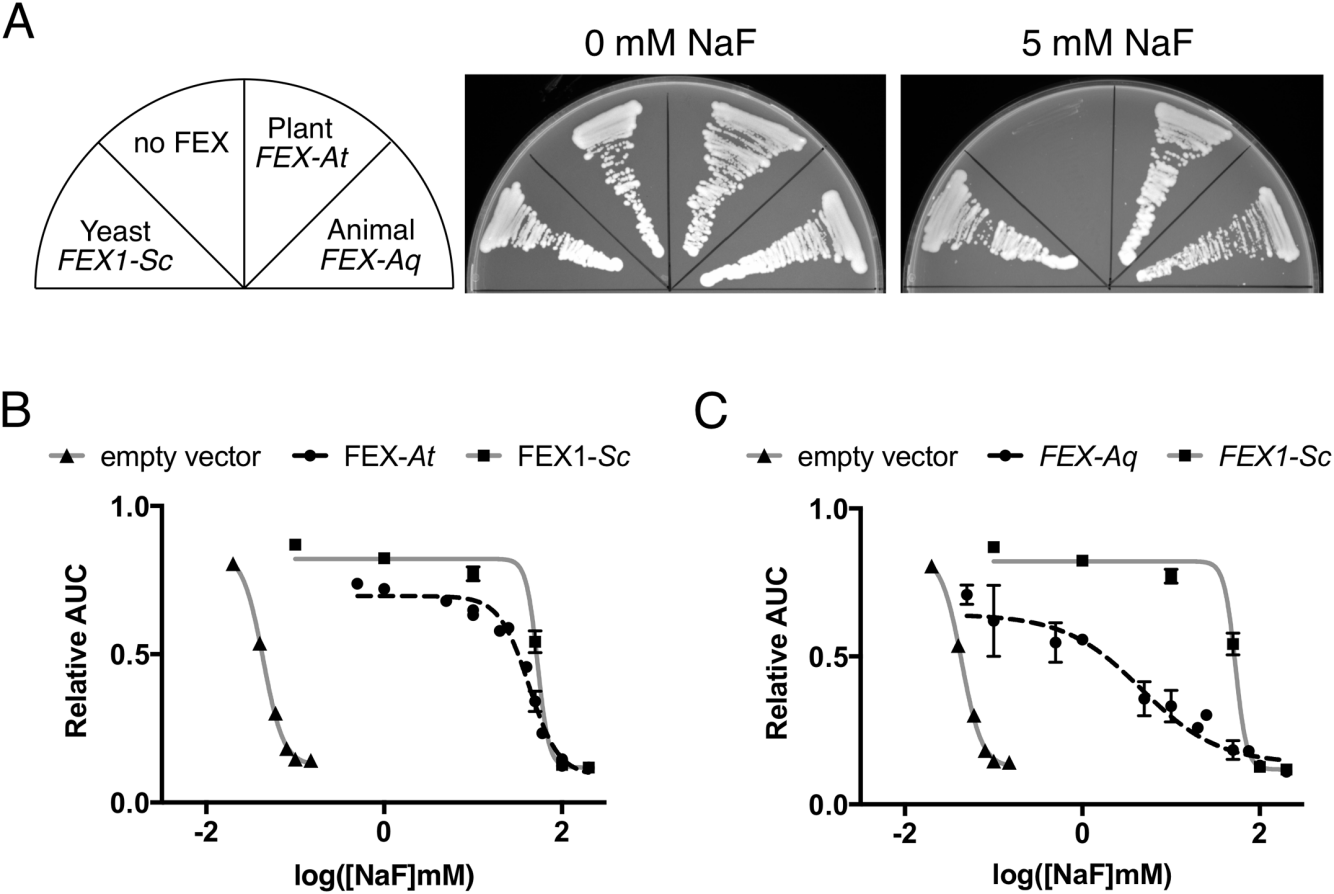
Growth rescue of *fex1Δfex2Δ* yeast expressing previously uncharacterized FEX-like genes. (A) The strains were grown on YPD and YPD containing 5 mM NaF to determine rescue phenotype. Plant *FEX-At* is a putative FEX gene from *A*. *thaliana* cloned into the p426GPD vector. Animal *FEX-Aq* is a putative FEX gene from *A*. *queenslandica* cloned into the p426GPD vector. Yeast rescue plasmid (pRS416-*FEX1*) was used as a positive control and empty vector (p426GPD) was used as a negative control. (B) Quantification of the growth tolerance to NaF of yeast strains described in A.

The FEX-like protein from the model plant *Arabidopsis thaliana* is currently annotated as ORF At2g41705.1. It has only 27% sequence identity to yeast FEX, but it includes all of the residues identified as important for fluoride tolerance. As in yeast, the key residues are located in Pore II and are not found in Pore I. We cloned the At2g41705.1 cDNA into a yeast expression vector with a constitutive glyceraldehyde-3-phosphate dehydrogenase (GPD) promoter that was used previously for functional expression of the bacterial ortholog in yeast. Expression of At2g41705.1 in the FEX double knock-out yeast strain rescued growth on NaF-containing media (Fig 6A). Qualitative measurement shows that the yeast strains expressing the plant gene grew as well on 5 mM NaF plates as yeast expressing its own FEX gene. In addition to the qualitative growth test, we quantified fluoride tolerance of yeast strains expressing At2g41705.1, which showed rescue similar to *FEX1* from *S*. *cerevisiae* (Fig 6B). Calculation of the fluoride dose response showed an IC_50_ of approximately 43 mM, which is more than a 1000-fold rescue relative to the empty vector control (Table 3).

**Table 3:** Growth tolerance of FEX-like genes from multicellular organisms relative to empty p426GPD vector.

Gene	Organism	Kingdom	IC_50_ (mM)	Fold rescue
p426GPD (empty vector)			0.033 ± 0.002	1
At2g41705.1	*A*. *thaliana*	Plant	43 ± 3	1300
105313411	*A queenslandica*	Animal	4.2 ± 3.6	130

FEX-like candidate genes can be found in several animals based upon the consensus sequence motifs identified above. All the candidates are found within aquatic organisms that live in the ocean. The sea sponge *Amphimedon queenslandica* has a FEX-like gene annotated as 105313411. The sequence identity of the 105313411 gene product to yeast FEX is 36%, which is relatively high for this family. As in plants, 105313411 has conserved residues only in Pore II and not Pore I. We cloned the 105313411 gene from *A*. *queenslandica* into the yeast vector as described above with a constitutive promoter. Expression of this clone in fluoride-sensitive yeast provided growth tolerance in the media containing 5 mM NaF (Fig 6A). Quantitative assay of the *A*. *queenslandica* gene 105313411 resulted in 10-fold lower tolerance to NaF then yeast FEX (Fig 6C). The resulting IC_50_ was 4.2 mM, which is a 130-fold rescue relative to the empty vector control (Table 3). Given the complexities of heterologous expression of foreign genes in yeast, greater than 100-fold rescue for both genes is a strong indication that these two previously unassigned genes are both functional FEX proteins.

## Discussion

**Fig 7:**
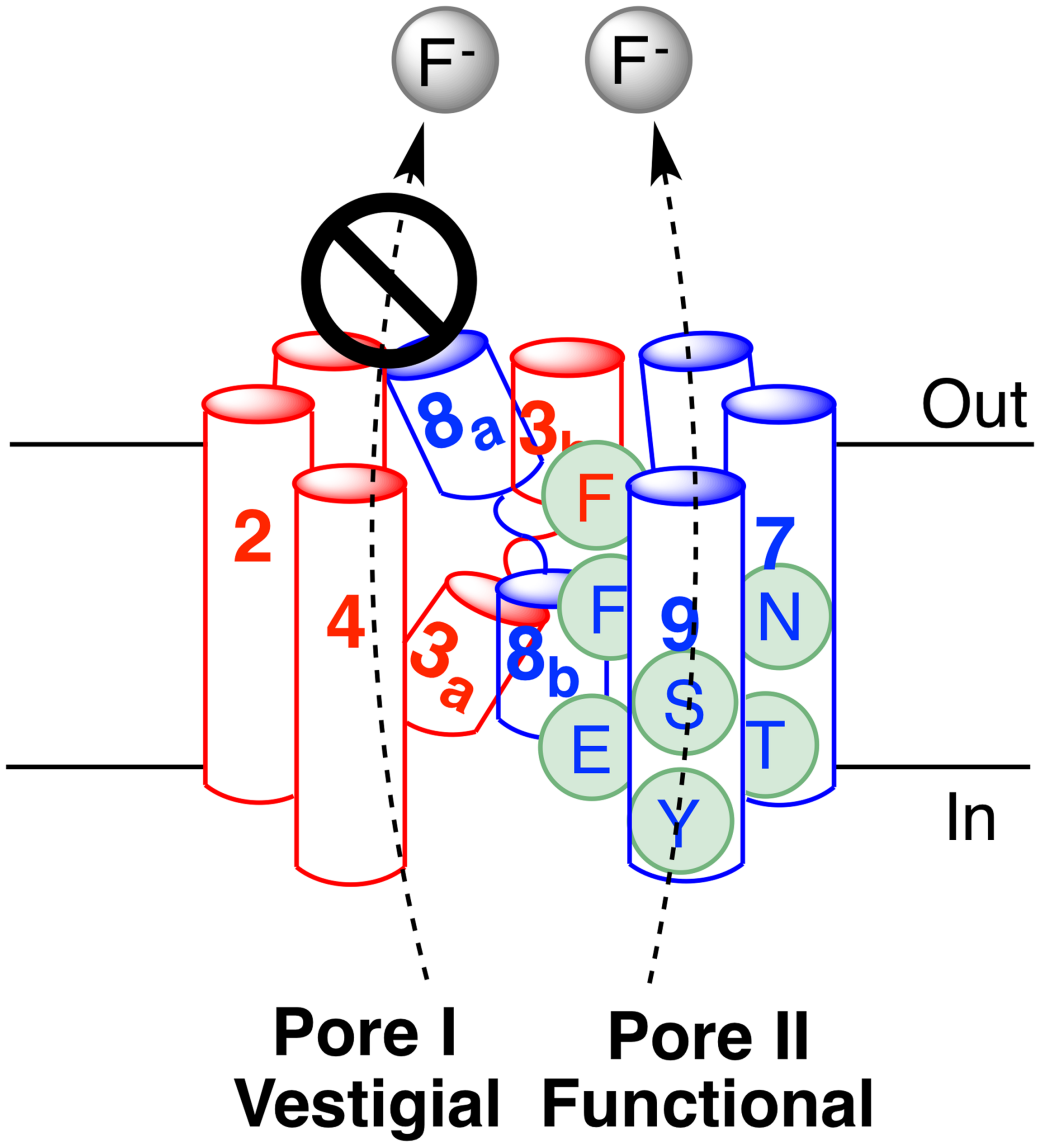
The helices from both domains that form functional and vestigial pores.

FEX proteins are important for fluoride tolerance in eukaryotes [16]. Baker’s yeast *S*. *cerevisiae* deficient in FEX are sensitive to low micromolar fluoride ion, a concentration that is common in the natural environment and many municipal water supplies. Expression of active FEX provides a detoxification mechanism allowing wild-type yeast to grow at a normal growth rate when exposed to low millimolar concentrations of fluoride [16]. Fluoride sensitivity of yeast to mutations in conserved residues provides an efficient means to explore their functional importance for channel activity [18]. Using a yeast growth assay we have determined how mutations of conserved motifs change activity of FEX. Here, we provide evidence that eukaryotic FEX proteins retain only one functional pore, in contrast to two functional pores in bacterial Fluc, yet the eukaryotic channel retains both domains. This suggests that one pore is active and another pore is vestigial in eukaryotic fluoride channels (Fig 7). In all proteins we could identify, Pore II was functional in fluoride transport, while natural evolutionary drift in Pore I had left it dysfunctional. In spite of this evolutionary degeneration of one of the pores, the overall architecture of the channel is conserved from bacteria to eukaryotes.

We have identified conserved motifs in the FEX proteins that include key residues that line the pore and residues that mediate interaction between the two protein domains. There are conserved hydrogen-bond donating and aromatic residues in all FEX proteins that match those found in Fluc [14]. These residues line Pore II and contribute to the Na^+^ binding site, but are not found in Pore I. Fluc is typically a homodimer and has equivalent motifs in both pores [14, 15]. Even in the case of *Lactobacillus acidophilus*, where one functional unit is formed by dimerization of two unique monomers, both Fluc-*La1* and Fluc-*La2* have these conserved motifs [12]. Bacterial Fluc proteins have two pores that have opposite orientations within the membrane [13–15]. Each pore transports fluoride ions and mutational inactivation of either pore caused only a two-fold decrease in the fluoride transport rate [15]. This suggests that each pore moves fluoride ions in the same direction despite the antiparallel orientation of the pores in the membrane. Our data suggest that eukaryotes retained only Pore II through the course of evolution. It is also possible that there is another subgroup of FEX proteins with a functional Pore I, though examples of such an arrangement were not evident in our bioinformatic analysis.

How is the FEX pore selective for fluoride? Physiological studies showed that FEX is at least 10-fold and Fluc is >10000-fold more selective for fluoride over chloride [12]. It is likely that FEX has higher than 10-fold ion selectivity [18] but it could not be measured accurately because of experimental limitations of whole-cell patch clamping of yeast. Our mutational studies of FEX, combined with physiological and structural studies of Fluc, provide insight into the mechanism of the anion selectivity within FEX. The high electronegativity and small atomic radius of fluoride makes it a good hydrogen bond acceptor. Many conserved residues with hydrogen bonding potential are predicted from the Fluc structure to line Pore II of FEX and are critical for fluoride tolerance in yeast. These residues can mimic the chemically preferred hydrated state for the fluoride ion, perhaps permitting the desolvation of fluoride from aqueous solution. A set of two highly conserved Phe residues is also important for the function of FEX. In the case of Fluc, the importance of these Phes was explained by the weak interactions with aromatic C-H that are most likely key elements for ion selectivity within this protein family [15]. In addition to the evidence from Fluc studies, fluoride ions have been shown to interact with aromatic rings and hydrogen-bond donors in the X-ray structures and NMR spectra of synthetic molecules [19].

The residues near the sodium binding site are conserved in Fluc and both domains of FEX. The flexible linkers splitting TM3a/b and TM8a/b allow the F96 side chain to turn toward Pore II. This two-helix crossover is probably assisted by a cation that was identified in Fluc, most likely Na^+^. Small soluble residues ooFooX/ooXooF that could be part of the organized hydrogen-bonding network around the cation are crucial for FEX activity in yeast. Mutations of these residues in N- and C-terminal domains made FEX protein inactive, which suggests the importance of this hydrogen bonding network.

Protection from fluoride toxicity is crucial for all domains of life. Homologs of FEX are found not only in unicellular organisms but also in multicellular eukaryotes. BLAST searches using *S*. *cerevisiae* FEX identified FEX homologs in important agricultural plants, such as corn, rice, grapes, oranges and cucumbers. If the search is restricted within the kingdom *Animalia*, the search returns three FEX homologs from aquatic species. This suggests that protection from fluoride is provided by a FEX homolog even in multicellular organisms. However, the FEX candidates have insufficient sequence identity to assign them as FEX, so further experimental evidence is necessary to conclude that they are indeed fluoride exporters. In fact, the sequence identity of FEX proteins between *S*. *cerevisiae* and *N*. *crassa*, two partially characterized examples, is only 23%, which is in the same range as the uncharacterized FEX-like proteins from other species. The ability of the FEX-like proteins to rescue growth of fluoride sensitive *S*. *cerevisiae* described in this study provides compelling evidence that ORFs similar to the FEX protein in *Arabidopsis thaliana* and sea sponge *Amphimedon queenslandica* serve as fluoride channels in these multicellular eukaryotes.

Plants also express a FEX-like protein. In fact, almost all plants for which a genome is available have a predicted open reading frame that encodes a FEX homolog. There are few studies about fluoride toxicity and accumulation in *Arabidopsis* and other plants [20–24]. It was reported that shoots and leaves absorb fluoride from gaseous HF in the air near some industrial enterprises, while roots uptake fluoride from water and soil where fluoride can exist naturally or as a result of water fluoridation [20]. One study using *Arabidopsis* cell suspension showed no defects in cell viability after treatment with 20 mM NaF for 2 h [25]. However, even 1 mM NaF inhibited cell growth after 10 days [25]. Some plants even have an ability to uptake fluoride from the soil and convert it to the even more toxic fluoroacetate as a deterrent to consumption by animals [26]. Fluoride accumulation has been detected in all parts of the plant with the anion most highly concentrated in the leaves [24]. Interestingly, examination of the relative expression of At2g41705.1 in *Arabidopsis* microarray data [27] visualized with the eFP browser [28] indicates higher expression in senescing leaves and dry seeds. It is possible that FEX in plants might aid in keeping the plant cells fluoride-free and F^-^ goes to the leaves leading eventually to abscission.

Aquatic animals are perhaps the most fluoride-exposed of the multicellular organisms. They are reported to accumulate fluoride in the exoskeleton of invertebrates and in the bones of fishes [29]. The rest of the fluoride is removed. The sea sponge *A*. *queenslandica* is a native of the Great Barrier Reef in the Coral Sea, off the coast of Queensland and its genome has been sequenced [30]. Interestingly, Queensland is one of a few places that limits water fluoridation, at least partially because of already substantial natural levels of fluoride. In some regions of Queensland, the fluoride concentration exceeds 130 μM (2.5 ppm) [31], a concentration at which the FEX-deleted yeast strain cannot grow. These data suggest that FEX is at least partially responsible for fluoride detoxification in some animals.

Despite fluoride ubiquity, there are eukaryotes that do not have a predicted *FEX*-like gene. There are many organisms that are exposed to fluoridated water and other fluoride enriched environment [20] that are not predicted to express FEX-like proteins. It is reasonable to expect that the necessity for fluoride detoxification extend beyond the species with an evident FEX-like ORF. Alternatively, there could be other mechanisms of fluoride tolerance, such as fluoride transport through another membrane bound protein, sequestration by proteins or in granule complexes, or a stronger cellular barrier from fluoride ions. It is also possible that a FEX gene exists in all eukaryotes, but the sequence identity of FEX becomes even lower than 20% in higher animals and thus is difficult to identify using current bioinformatic search mechanisms. The low number of animals with sequenced genomes, as well as the splicing variations of possible transcripts, could contribute to a misleadingly low distribution of FEX in higher eukaryotes. The sequence motifs identified in the current study might help find yet unidentified FEX proteins that possibly exist in animals by providing additional criteria in searching algorithms.

## Materials and methods

### Reagents

Sodium fluoride was purchased from Sigma-Aldrich. Bacto Yeast Extract, Bacto Peptone, Bacto Agar, and Difco yeast nitrogen base without amino acids were purchased from BD Biosciences. Glucose (dextrose) was purchased from J. T. Baker. All chemicals were >98% pure according to the manufacturer quality controls.

### Yeast strains

All *S*. *cerevisiae* strains and primers are summarized in Table A and Table B in S1 File. The parent strain used for all transformations is SSY3, which is a derivative of BY4741 strain, that has both FEX genes deleted (*MATa his3Δ1 leu2Δ0 ura3Δ0 FEX1Δ*::*kanMX6 FEX2Δ*::*hphMX4*). Yeast strains were maintained routinely on YPD or SD-ura medium. Yeast cultures were grown overnight by agitation (220 rpm) at 30°C prior to each experiment.

### Site-directed mutagenesis of *FEX1*

Mutagenesis was performed with pRS416-*FEX1* rescue plasmid [18] as a template using the QuickChange II site-directed mutagenesis protocol (Agilent Technologies). Briefly, primers (Keck Oligonucleotide Synthesis Facility) were designed with the desired mutation flanked by bases of perfect homology to plasmid template on each side. After PCR the template was digested with *DpnI* restriction enzyme (New England Biolabs) and the product was transformed into chemically competent MAX Efficiency *DH5a* Competent Cells (Thermo Fisher Scientific). Plasmids carrying the targeted mutation(s) were isolated using GeneJet Plasmid Miniprep Kit (Thermo Fisher Scientific) and then verified by DNA sequencing (Keck DNA Sequencing Facility) using primers specific to the upstream and downstream regions of FEX gene (see Table B in S1 File for details).

### Growth measurements

Confirmed plasmids were transformed into *fex1Δfex2Δ* yeast (strain SSY3 S1 File) using the standard lithium acetate protocol. Transformed strains were selected on SD-ura plates at 30°C for 48 hr. For liquid growth assays, overnight cultures were diluted to a final OD_600_ of 0.1 in 24-well plate (Costar) in YPD with different amounts of NaF to a total volume of 1 mL per well. The assays were performed in a Biotek Synergy plate reader at 30°C with continuous agitation. Absorbance values were recorded every 3 min. Curves were plotted as absorbance change over time and analyzed using GraphPad Prism. To determine IC_50_ values, the area under the curve was calculated for each growth curve. These values were normalized relative to the growth curve without fluoride. The normalized values were plotted vs. the log of the fluoride concentration and fit to a standard dose–response curve with GraphPad Prism. The values are summarized in Tables 1, 2 and 3.

Yeast spot assay was performed from the overnight culture by adjusting OD_600_ = 1 and making a 10-fold dilution series in sterile water. Approximately, 5 μL of culture was plated on YPD or YPD + NaF agar plates and incubated for 48 h at 30°C before imaging.

### Cloning of FEX *Arabidopsis thaliana* and *Amphibedon queenslandica*

The putative *Arabidopsis thaliana* FEX ORF (At2g41705.1) was amplified with Phusion High Fidelity polymerase (NEB) from cDNA made from mature leaves of col-0. plants grown on soil (RETROscript, Thermo Fisher). The resulting DNA was cloned into TA vector 2.1, sequence verified and re-amplified with primers that included 20 bases of the beginning and end of the *A*. *thaliana* FEX ORF and 45 bp of homology to the p426GPD vector upstream and downstream of *HindIII* restriction site. Sequence of FEX gene from *A*. *queenslandica* (UPF0695 membrane protein, see S1 File for gene sequence) was synthesized by GeneArt, Thermo Fisher Scientific Inc. The ends of both fragments contained 45 bp of homology to the p426GPD vector backbone upstream and downstream of *HindIII*. Fragments were transformed with *HindIII* linearized p426GPD vector into SSY3 according to high-efficiency transformation protocol. Strains that successfully integrated inserts into vector were selected on SD-ura media, their DNA was isolated, transformed into DH10B cells, and sequence verified by the Keck DNA Sequencing Facility. Both sequence-verified constructs and positive (pRS416-*FEX1*-*Sc*) and negative (pRS416 empty vector) controls were transformed to SSY3 strain and tested for growth by streaking on YPD plate with 5 mM NaF. A control plate contained YPD only.

### Structural modeling of Fex1p

The eukaryotic fluoride channel structure was modeled using the crystal structure of Fluc protein from *Bordetella pertussis* with the highest resolution (2.17 Å) (PDB ID: 5FXB) that allows identification of the fluoride ions. The Fex1p structure was built using SWISS-MODEL workspace with Target-Template Alignment [32]. Sequence alignments for structural modeling of Fluc-*Bp* to N-terminal domain (from L12 to I147) and Fluc-*Bp* to C-terminal (from S237 to C375) domain were generated with Clustal Omega [33]. Generated structural models of two Fex1p domains were aligned to the crystal structure of two antiparallel Fluc-*Bp* domains (chains A and B of 5FXB) using PYMOL. Coordinates of fluoride and sodium ions from the crystal structure of Fluc-Bp (chains A and B) were used to estimate location of these ions in Fex1p.

### Protein sequence alignment

FEX-like protein sequences were determined with BLAST search using Fex1p from *S*. *cerevisiae*. All together 190 proteins were included into alignment file. Out of these 190 proteins 3 belonged to animals, 76 to plants and the rest to fungi. Transmembrane helices were predicted using TMHMM2 v2.0 [34] and TMpred servers. Sequences of each TMH region from bacterial and eukaryotic exporters were aligned with Clustal Omega [33]. Alignment files were colored by sequence similarity using ESPript 3.0 [35] to highlight conserved residues in each TMH.

## Supporting information

S1 Fig Conservation of TM8 sequence in FEX-like proteins. (TIFF)

S2 Fig Expression analysis of Fex1p. Western blot analysis of yeast expressing FEX1 wild-type protein and mutants tagged with HA tag. PMA1 was stained as a control for plasma membrane proteins.
(TIFF)

S3 Fig Growth curve of yeast expressing FEX1 protein with mutations A49P:P50A:T51G:V52T:F96A:M99F:I327F:F330A. (TIFF)

S1 File Supplementary file. List of strains, primers and growth curve data.
(PDF)
