# Supplementary figures and images for: Fluoride export (FEX) proteins from fungi, plants and animals are 'single barreled' channels containing one functional and one vestigial ion pore
Source: PLoS One. 2017 May 4;12(5):e0177096. doi: 10.1371/journal.pone.0177096 (PMC5417652; doi:10.1371/journal.pone.0177096)

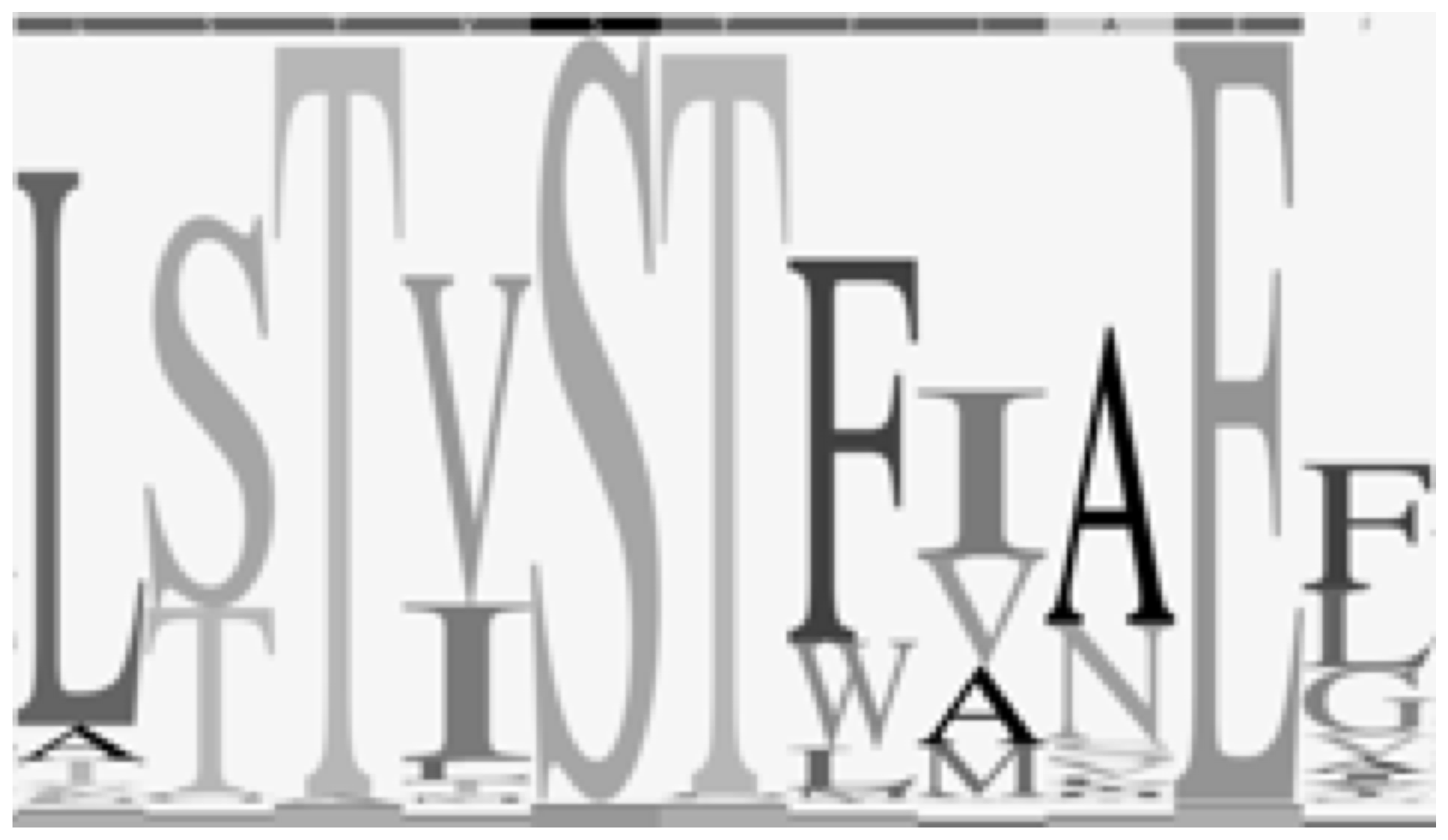

Supplement: S1 Fig — (TIFF) [file pone.0177096.s001.tiff]

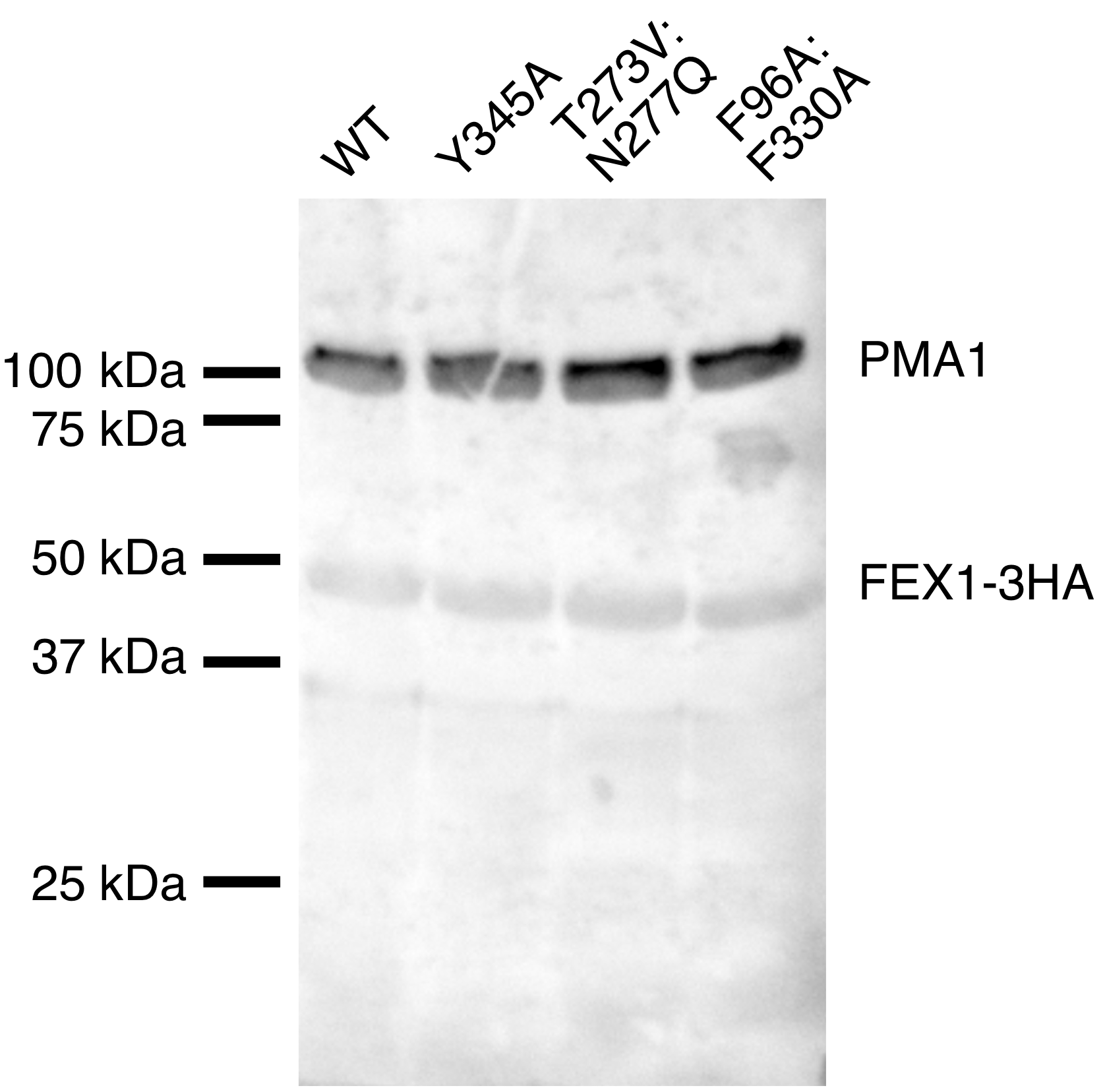

Supplement: S2 Fig — Western blot analysis of yeast expressing FEX1 wild-type protein and mutants tagged with HA tag. PMA1 was stained as a control for plasma membrane proteins. (TIFF) [file pone.0177096.s002.tiff]

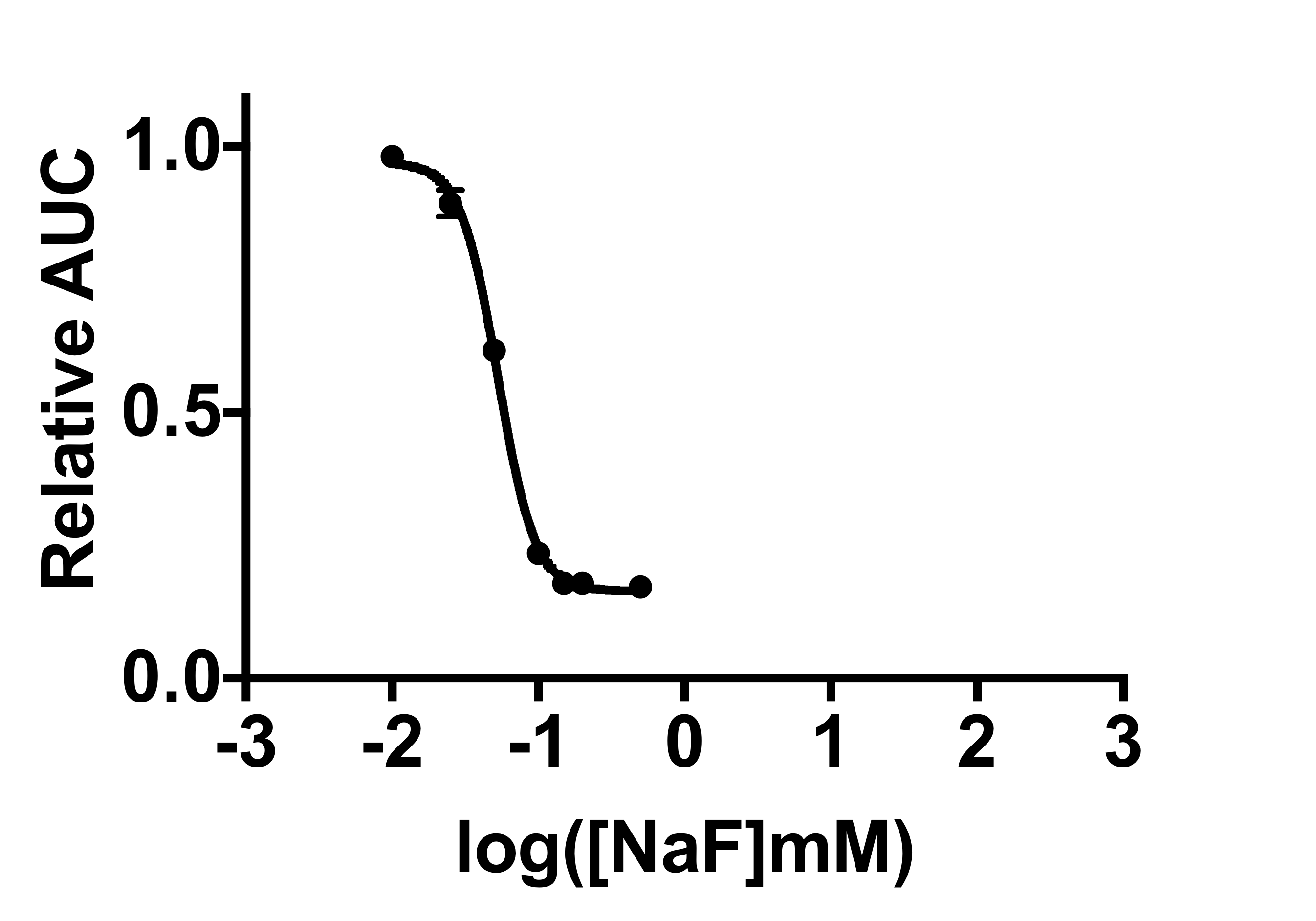

Supplement: S3 Fig — (TIFF) [file pone.0177096.s003.tiff]
