# Supplementary material for: Fluoride export (FEX) proteins from fungi, plants and animals are 'single barreled' channels containing one functional and one vestigial ion pore
Source: PLoS One. 2017 May 4;12(5):e0177096. doi: 10.1371/journal.pone.0177096 (PMC5417652; doi:10.1371/journal.pone.0177096)
Supplement: S1 File — List of strains, primers and growth curve data. (PDF) [file pone.0177096.s004.pdf]

Supplemental Information for:

Fluoride export (FEX) proteins from fungi, plants and animals are ‘single barreled’ channels containing one functional and one vestigial ion pore

Tetyana Berbasova[1,2], Sunitha Nallur[1,2], Taylor Sells[1,2], Kathryn D. Smith[1,2], Patricia B. Gordon[3], S. -Lori Tausta[1,2], Scott A. Strobel[1,2]*.

[1]Department of Molecular Biophysics and Biochemistry, Yale University, New Haven, CT, USA

[2]Chemical Biology Institute, Yale University, West Haven, CT, USA

[3]Division of Basic Sciences, Fred Hutchinson Cancer Research Center, Seattle, WA, USA

*To whom correspondence should be addressed. Phone: (203) 432-9772. Fax: (203) 432-5767. Email: scott.strobel@yale.edu.

Supplemental Tables

Table A. Yeast strains used in this study. All strains were made in this study unless noted.

Name	Description	Genotype
SSY**3**[a]	*fex1Δ fex2Δ*	*MAT***a** *his3Δ1 leu2Δ0 ura3Δ0 fex1Δ::kanMX6 fex2Δ::hphMX4*
SSY**5**[a]	pRS416-*FEX1* (rescue strain)	*MAT***a** *his3Δ1 leu2Δ0 ura3Δ0 fex1Δ::kanMX6 fex2Δ::hphMX4* [pRS416-*FEX1*]
SSY**49**[b]	N277Q	*MAT***a** *his3Δ1 leu2Δ0 ura3Δ0 fex1Δ::kanMX6 fex2Δ::hphMX4* [pRS416-*FEX1* (N277Q)]
SSY**57**	F96A	*MAT***a** *his3Δ1 leu2Δ0 ura3Δ0 fex1Δ::kanMX6 fex2Δ::hphMX4* [pRS416-*FEX1* (F96A)]
SSY**58**	F330A	*MAT***a** *his3Δ1 leu2Δ0 ura3Δ0 fex1Δ::kanMX6 fex2Δ::hphMX4* [pRS416-*FEX1* (F330A)]
SSY**59**	F96A:F330A	*MAT***a** *his3Δ1 leu2Δ0 ura3Δ0 fex1Δ::kanMX6 fex2Δ::hphMX4* [pRS416-*FEX1* (F96A:F330A)]
SSY**60**	F96A:F330A:M99F:I327F	*MAT***a** *his3Δ1 leu2Δ0 ura3Δ0 fex1Δ::kanMX6 fex2Δ::hphMX4* [pRS416-*FEX1* (F96A:F330A:M99F:I327F)]
SSY**61**	P270A	*MAT***a** *his3Δ1 leu2Δ0 ura3Δ0 fex1Δ::kanMX6 fex2Δ::hphMX4* [pRS416-*FEX1* (P270A)]
SSY**62**	T273V	*MAT***a** *his3Δ1 leu2Δ0 ura3Δ0 fex1Δ::kanMX6 fex2Δ::hphMX4* [pRS416-*FEX1* (T273V)]
SSY**63**	T273V:N277Q	*MAT***a** *his3Δ1 leu2Δ0 ura3Δ0 fex1Δ::kanMX6 fex2Δ::hphMX4* [pRS416-*FEX1* (T273V:N277Q)]
SSY**64**	E102A	*MAT***a** *his3Δ1 leu2Δ0 ura3Δ0 fex1Δ::kanMX6 fex2Δ::hphMX4* [pRS416-*FEX1* (E102A)]
SSY**65**	E333A	*MAT***a** *his3Δ1 leu2Δ0 ura3Δ0 fex1Δ::kanMX6 fex2Δ::hphMX4* [pRS416-*FEX1* (E333A)]
SSY**66**	E102A:E333A	*MAT***a** *his3Δ1 leu2Δ0 ura3Δ0 fex1Δ::kanMX6 fex2Δ::hphMX4* [pRS416-*FEX1* (E102A:E333A)]
SSY**67**	Y345A	*MAT***a** *his3Δ1 leu2Δ0 ura3Δ0 fex1Δ::kanMX6 fex2Δ::hphMX4* [pRS416-*FEX1* (Y345A)]
SSY**68**	Y345F	*MAT***a** *his3Δ1 leu2Δ0 ura3Δ0 fex1Δ::kanMX6 fex2Δ::hphMX4* [pRS416-*FEX1* (Y345F)]
SSY**69**	S349A	*MAT***a** *his3Δ1 leu2Δ0 ura3Δ0 fex1Δ::kanMX6 fex2Δ::hphMX4* [pRS416-*FEX1* (S349A)]
SSY**70**	S349V	*MAT***a** *his3Δ1 leu2Δ0 ura3Δ0 fex1Δ::kanMX6 fex2Δ::hphMX4* [pRS416-*FEX1* (S349V)]
SSY**71**	S94V:S95V	*MAT***a** *his3Δ1 leu2Δ0 ura3Δ0 fex1Δ::kanMX6 fex2Δ::hphMX4* [pRS416-*FEX1* (S94V:S95V)]

SSY**72**	S97V:S98V	*MAT***a** *his3Δ1 leu2Δ0 ura3Δ0 fex1Δ::kanMX6 fex2Δ::hphMX4* [pRS416-*FEX1 (*S97V:S98V*)*]
SSY**73**	S325V:T326V	*MAT***a** *his3Δ1 leu2Δ0 ura3Δ0 fex1Δ::kanMX6 fex2Δ::hphMX4* [pRS416-*FEX1 (*S325V:T326V*)*]
SSY**74**	S328V:T329V	*MAT***a** *his3Δ1 leu2Δ0 ura3Δ0 fex1Δ::kanMX6 fex2Δ::hphMX4* [pRS416-*FEX1 (*S328V:T329V*)*]
SSY**75**	S94V:S95V:S328V:T329V	*MAT***a** *his3Δ1 leu2Δ0 ura3Δ0 fex1Δ::kanMX6 fex2Δ::hphMX4* [pRS416-*FEX1 (*S94V:S95V:S328V:T329V*)*]
SSY**76**	FEX-*At*	*MAT***a** *his3Δ1 leu2Δ0 ura3Δ0 fex1Δ::kanMX6 fex2Δ::hphMX4* [p426GPD-*FEX-A.thaliana*]
SSY**77**	FEX-*Aq*	*MAT***a** *his3Δ1 leu2Δ0 ura3Δ0 fex1Δ::kanMX6 fex2Δ::hphMX4* [p426GPD-*FEX-A.queenslandica*]
SSY**78**	F96A:F330A – 3HA	*MAT***a** *his3Δ1 leu2Δ0 ura3Δ0 fex1Δ::kanMX6 fex2Δ::hphMX4* [pRS416-*FEX1*-3HA *(*F96A:F330A*)*]
SSY**79**	Y345A – 3HA	*MAT***a** *his3Δ1 leu2Δ0 ura3Δ0 fex1Δ::kanMX6 fex2Δ::hphMX4* [pRS416-*FEX1*-3HA *(*Y345A*)*]
SSY**80**	T273V:N277Q – 3HA	*MAT***a** *his3Δ1 leu2Δ0 ura3Δ0 fex1Δ::kanMX6 fex2Δ::hphMX4* [pRS416-*FEX1*-3HA *(*T273V:N277Q*)*]
SSY**81**	N-terminal domain (1-165)	*MAT***a** *his3Δ1 leu2Δ0 ura3Δ0 fex1Δ::kanMX6 fex2Δ::hphMX4* [p415GPD-*FEX1 (1-165)*]
SSY**82**	C-terminal domain (165-375)	*MAT***a** *his3Δ1 leu2Δ0 ura3Δ0 fex1Δ::kanMX6 fex2Δ::hphMX4* [p426GPD-*FEX1 (165-375)*]
SSY**83**	N-/C-terminal domains	*MAT***a** *his3Δ1 leu2Δ0 ura3Δ0 fex1Δ::kanMX6 fex2Δ::hphMX4* [p415GPD-*FEX1 (1-165)* p426GPD-*FEX1 (165-375)*]

[a]From (Li et al. 2013). [b]From (Smith et al. 2015).

Table B. Primers for site-directed mutagenesis.

Mutation in Fex1p-*Sc*	Sequence
F96A forward	GTGCCCTGTCTTCCGCTTCTAGTATGCTGC
F96A reverse	GCAGCATACTAGAAGCGGAAGACAGGGCAC
F330A forward	CTTTGAGTACCATTAGCACGGCTATCAATGAAGGGTATAAG
F330A reverse	CTTATACCCTTCATTGATAGCCGTGCTAATGGTACTCAAAG
F96A:M99F forward	GTGCCCTGTCTTCCGCTTCTAGTATGCTGC
F96A:M99F reverse	GCAGCATACTAGAAGCGGAAGACAGGGCAC
I327F:F330A forward	CTGTCTTCCGCTTCTAGTTTTCTGCTAGAGATGTTCGAAC
I327F:F330A reverse	GTTCGAACATCTCTAGCAGAAAACTAGAAGCGGAAGACAG
E102A forward	GCTGCTAGCGATGTTCGAACAC
E102A reverse	GTGTTCGAACATCGCTAGCAGC
E333A forward	CGTTTATCAATGCAGGGTATAAGCTATC
E333A reverse	GATAGCTTATACCCTGCATTGATAAACG

P270A forward	CAAACAAAAAGTTTGCATTGGGTACTTTC
P270A reverse	GAAAGTACCCAATGCAAACTTTTTGTTTG
T273V forward	GTTTCCATTGGGTGTTTTCTTGGC
T273V reverse	GCCAAGAAAACACCCAATGGAAAC
Y345A forward	CATGCTTATTGCTTATACTGTTTCGATTGC
Y345A reverse	GCAATCGAAACAGTATAAGCAATAAGCATG
Y345F forward	CATGCTTATTTTTTATACTGTTTCGATTGC
Y345F reverse	GCAATCGAAACAGTATAAAAAATAAGCATG
S349A forward	CTTATTTATTATACTGTTGCGATTGCAATTTC
S349A reverse	GAAATTGCAATCGCAACAGTATAATAAATAAG
S349V forward	CTTATTTATTATACTGTTGTGATTGCAATTTC
S349V reverse	GAAATTGCAATCACAACAGTATAATAAATAAG
S94V:S95V forward	CTGGGTACTGTGGTGCCCTGGTTGTCTTTTCTAGTATGCTGCTAG
S94V:S95V reverse	CTAGCAGCATACTAGAAAAGACAACCAGGGCACCACAGTACCCAG
S97V:S98V forward	GTGGTGCCCTGTCTTCCTTTGTTGTTATGCTGCTAGAGATGTTCG
S97V:S98V reverse	CGAACATCTCTAGCAGCATAACAACAAAGGAAGACAGGGCACCAC
S325V:T326V forward	GATTTTGCGGCACTTTGGTTGTCATTAGCACGTTTATC
S325V:T326V reverse	GATAAACGTGCTAATGACAACCAAAGTGCCGCAAAATC
S328V:T329V forward	GCACTTTGAGTACCATTGTCGTGTTTATCAATGAAGGG
S328V:T329V reverse	CCCTTCATTGATAAACACGACAATGGTACTCAAAGTGC
Sequencing primer, for	GAGATACAGAAGAGATACGG
Sequencing primer, rev	GTCCAAGCATAGGATCCGAGAG

At2g41705.1 forward	GATTCTAGAACTAGTGGATCCCCCGGGCTGCAGGAATTCGATATC AATGGATACGGGTCAAAGCAGCATAGAAC
At2g41705.1 reverse	GCGTGACATAACTAATTACATGACTCGAGGTCGACGGTATCGATA CTAACTGAATCCTATTACCCAGACAGG
105313411 forward	GATTCTAGAACTAGTGGATCCCCCGGGCTGCAGGAATTCGATATC ATGTCGTCCATTATAAGTGATTCTTATG
105313411 reverse	GCGTGACATAACTAATTACATGACTCGAGGTCGACGGTATCGATA TTAATAAACAACTGAATAAATAATGAATATT

DNA sequence of At2g41705.1 from *A. thaliana* cloned into p426GPD vector

ATGGATACGGGTCAAAGCAGCATAGAACCTTATCAAGCCAAGTCTTTTAGTCGGGAGAGCAG
TGTAGCCTCTTCTTTAAGCTTATCACGTAGTTTGCCTCACCTAATTGACAATGACGTCGATAG
TGAGAGTGTCTCAGAGGCAGGGGATATTGGGGACCGCTCACTTCGGAGAAGGCATAGTGCT
GGTAGAAGCAGCCGTTTGTCTGCTGATGATTTTATAGAACAAGGGACTCATGATACTTCTCG
TCAAGAACAAGATATATTACATGACCTTCGAGCTTTCAACACTGCTTCCGTAAATAAAACTTT
GCCTGAGGACATAACAGCATCTCCTTTACCGACCAAGTCACTCTTGTCACCTGAAATAAACA
ACTCCGGAAAGGAGGAAGAGCGAGTGTTACCAAAGTCCTTGGAGTACATATCATGCCTAATT
CATTTGGCTGTTTTTGGGATTTTTGGGGCCATTACGAGATATTTGCTGCAAAAATTGTTTGGG
CCAACTGGTGCTCGAGTAACAAGTGATGGGAGCATCTTGTACCTTGATCTTCCCTCCAACAT
GGTAGGATCATTCTTGATGGGTTGGTTTGGCGTTGTATTCAAAGCAGATATAGCAAGAGTTT
CTGAATTTGTGGCGATAGGATTATCGACTGGTTATTTGGGGAGTCTGACAACATTCAGCGGT
TGGAACCAGAAAATGCTGGATCTTAGTGCTGATGGTCAATGGGTGTATGCTGTGCTTGGCTT
TTTATTAGGATTGTTTCTCACGTCATACTCCATAATTCTGGGAGTGGAAACGGCCAAAGGGTT
TAAATGGCTTCTTCATAGAAGAGCTTCTTCTGAGGATAAATTCCATTGTCTTAAGGTTAACAC
CTTCCAGAGCCATATTGTGTCTCTGACCCTGATGCTTCTGTTGCTTGTGGCTTTACTCACTGC
CAGTTCCATACTGCTTGTGAAAGAGTTTGACAAAGGAACAAGCGAGGCTCAGCTATGGTTTG
GTTGCTTAGTTGCAGCCCCTGGTGTCTGGCTCAGATGGTTCTTAGCCCGACTCAATGGACGT
GGGCTGGGAAAGGATAGTCAAAATTTGAGATGGGTCCCATTTGGCACCCTCATTGCAAATGT

AGTTGCAGCTTGCGTTATGGCAGCATTGGCTACCTTGAAGAAATCGGTGAACACGAGAACAT
GTAACACGGTTGCTTCGAGCATACAGTTTGGTCTGTTGGGATGTCTGAGCACAGTTTCGACC
TTCATGGCTGAGTTCAATGCGATGAGAGAAAGTGATTACCCATGGAGAGCCTATGCGTATGC
ATCTTTTACCATTGTGGTTTCTTTTGCGATTGGAACTATTATATACTCAGTCCCTGTCTGGGTA
ATAGGATTCAGTTAG

DNA sequence of 105313411 from *A. queenslandica* cloned into p426GPD vector:

ATGTCGTCCATTATAAGTGATTCTTATGGTGCAACAGAATTACCAGACAGTTCTGATTCCAGT
GATACCGTTCTTGATGTTACAGCTGATGACAAGCCAAGAGATGGTAATTGTTACAAGTACCTT
CATCATTTCCTAATGATAGTGTGTTTATCAATTGGGTCTTATCTTGGTGTCATCACAAGGATAT
ATACCTCAATATATTTATCATTATTTGATAATATCGATCAGTTTACATCTCTCTGGGCTCAAGT
TATAGGTACTGGAGTCATTGGGTATCTAGTCATCAATAAAAGTAACATTAATAATGTATTGTAC
ACTTCACTGGCCACAGGACTGTGTGGCTCACTCACCACATTTTCAACATGGAATGCTGAGTC
AGCATTGGTGCTGCTCCAGTTGAACGAGTCTACTCTTGTTACTATAAATAGACCAGACTACGT
TAAAGGTGGAGTGTCAGGTCTCATGATACTATTTATAGGTATTGGTCTACCTTTGTCATCATT
TATATTTGGTAGCAATATGGGACGGGTATTCAAAGCACCTGTAATTACCAGTAGCTATTATGT
CACTGGATATGTTAGCTCAGTGTTGTTGTACATAATAAGTACAACAATCATCATTATTGTCTGT
TTAATTACTGATAATTATTATATTCTTTTTTCACTTTTATTTGGTCCAATTGGCACTTATTTGAG
GTGGCGATTAGCTTATTTTGATGTTAACTTTCTTAAAAACGATTTTCCAATGGGAACATTGATA
TCTAATTACACTGGATCACTGATATTAGCAGGTTGTATGGTTGCCAGGTTACACGTCAACAAT
CCAACTGTAGTTCAGTTAATAAATGGAGTCATTACTGGTTTCTGTGGCTGCTTGACAACAGTT
TCAACATTCATCAGTCAAATAACCAAACTCTCATTCAAAATGTCATCAATATATGTACTAATAT
CACTGACTACAGTACAAATAACTTACATTACAATATTCATTATTTATTCAGTTGTTTATTAA

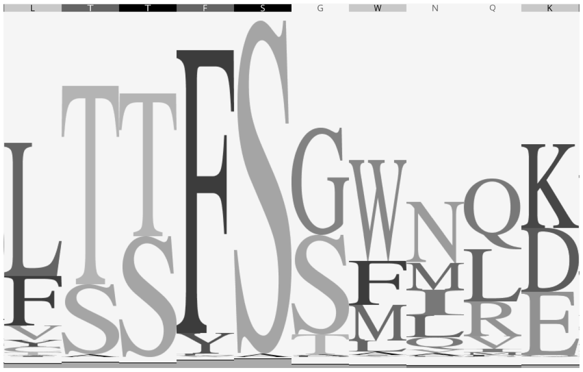

Figure A. Conservation of TM3 sequence in FEX-like proteins.

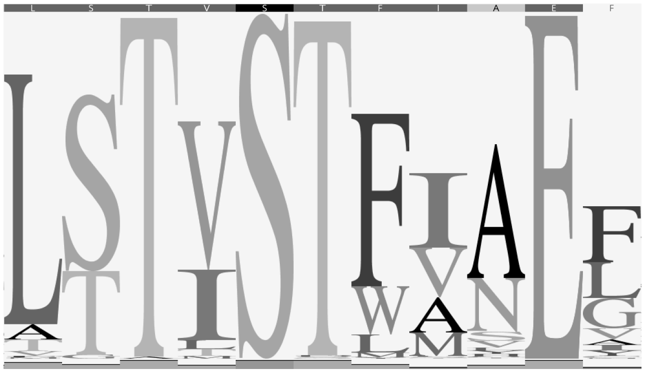

Figure B. Conservation of TM8 sequence in FEX-like proteins.

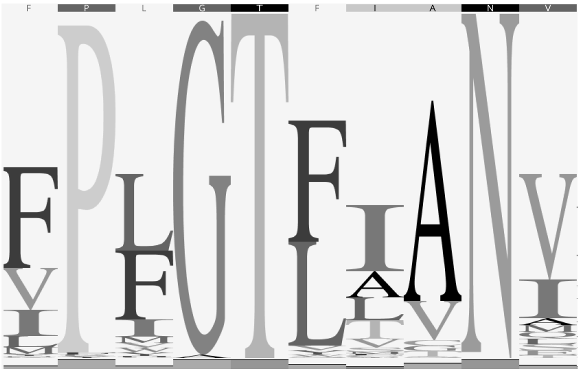

Figure C. Conservation of TM7 sequence in FEX-like proteins.

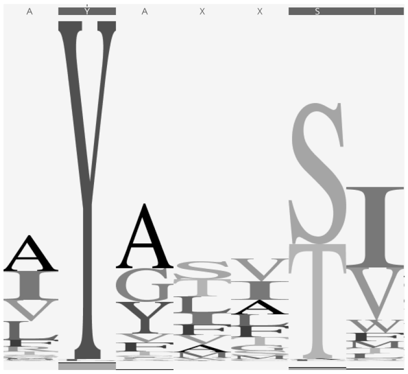

Figure D. Conservation of TM9 sequence in FEX-like proteins.

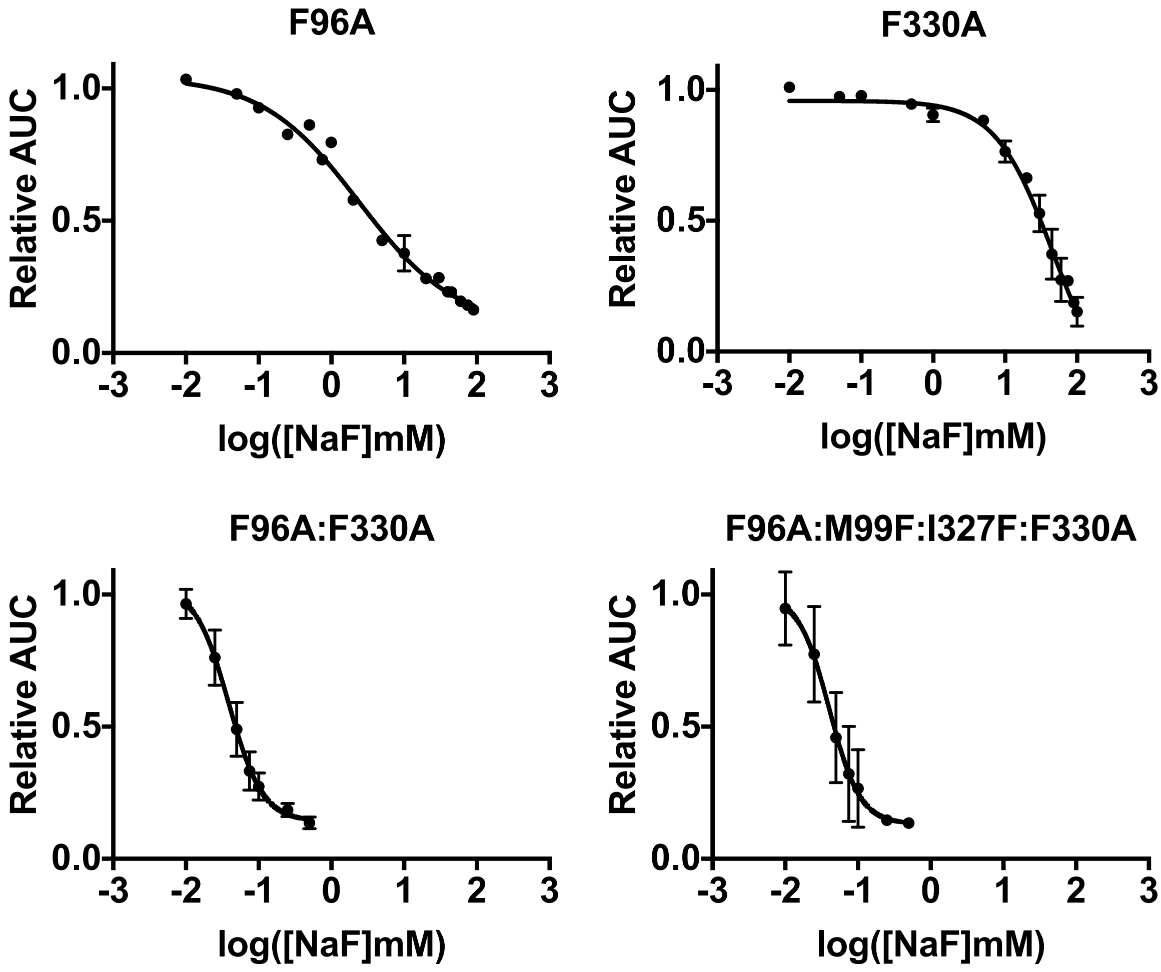

Figure E. Growth curves of yeast expressing FEX proteins with mutations at conserved phenylalanine residues.

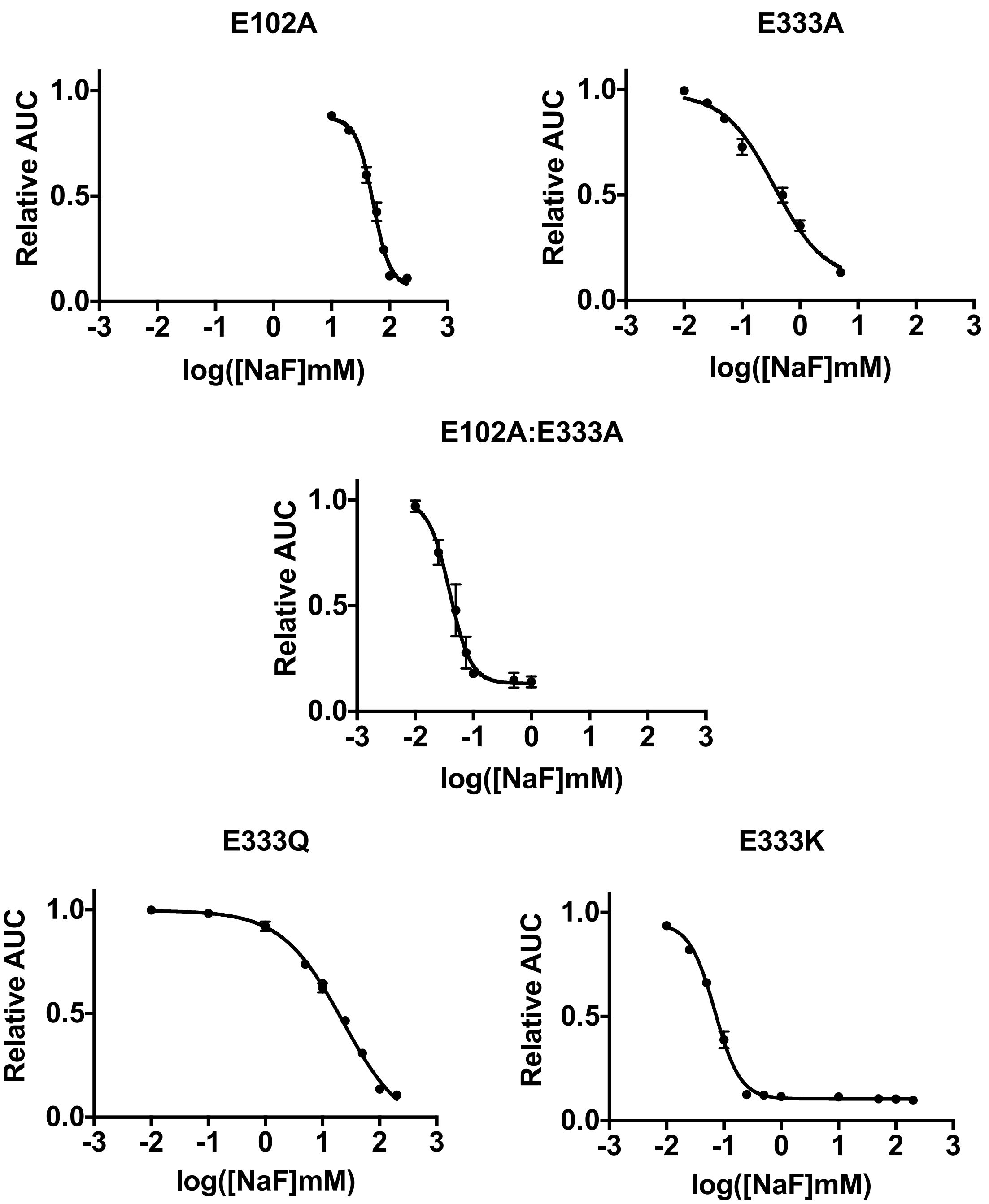

Figure F. Growth curves of yeast expressing FEX proteins with mutations at E102 and E333.

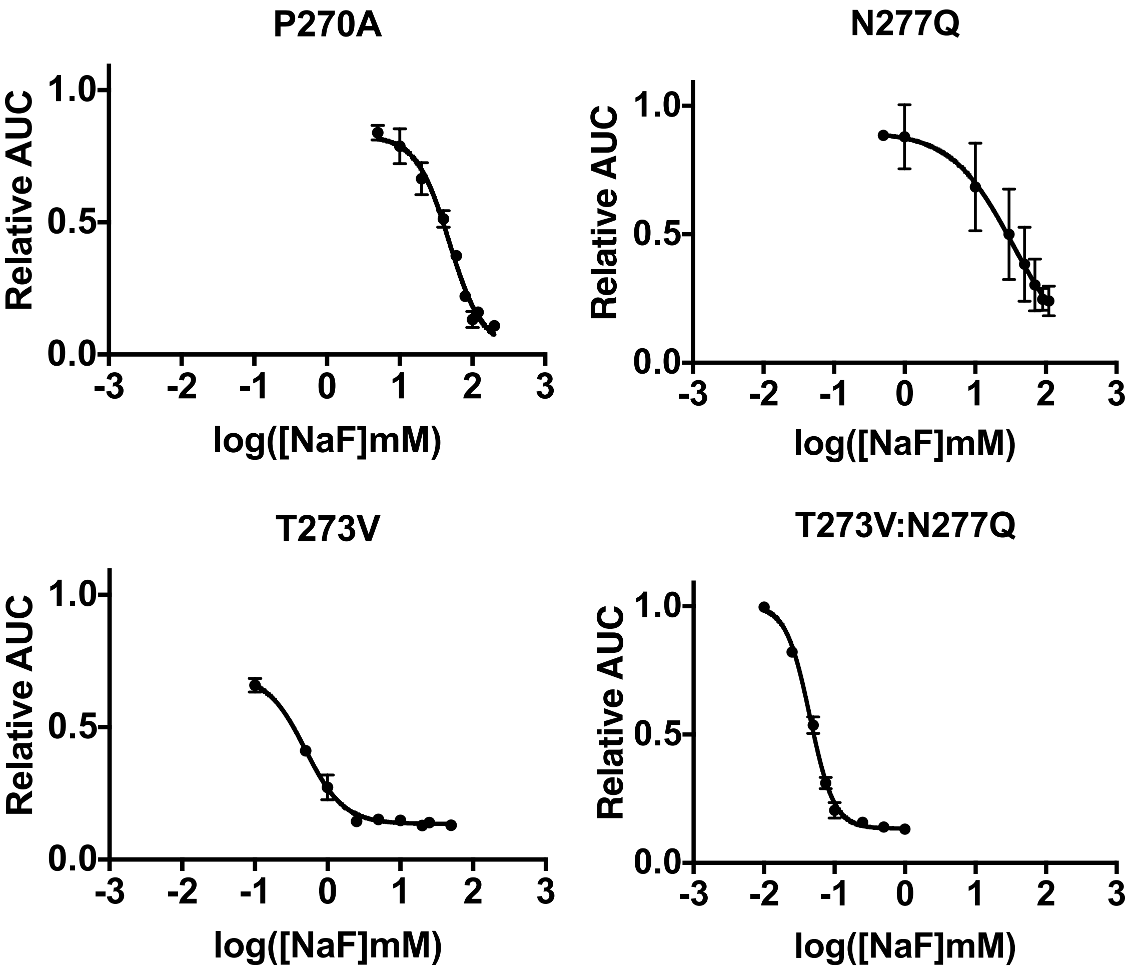

Figure G. Growth curves of yeast expressing FEX proteins with mutations at conserved PxGTxxxN motif.

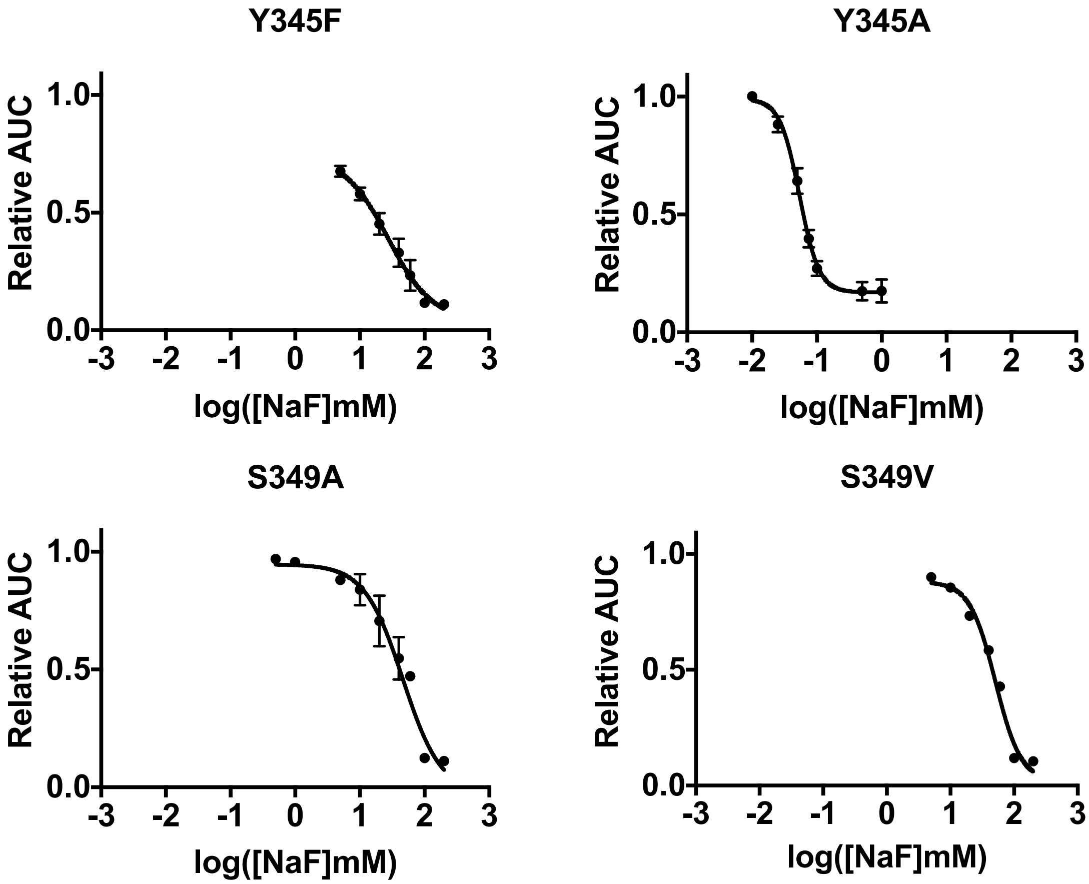

Figure H. Growth curves of yeast expressing FEX proteins with mutations at conserved YxxxS motif.

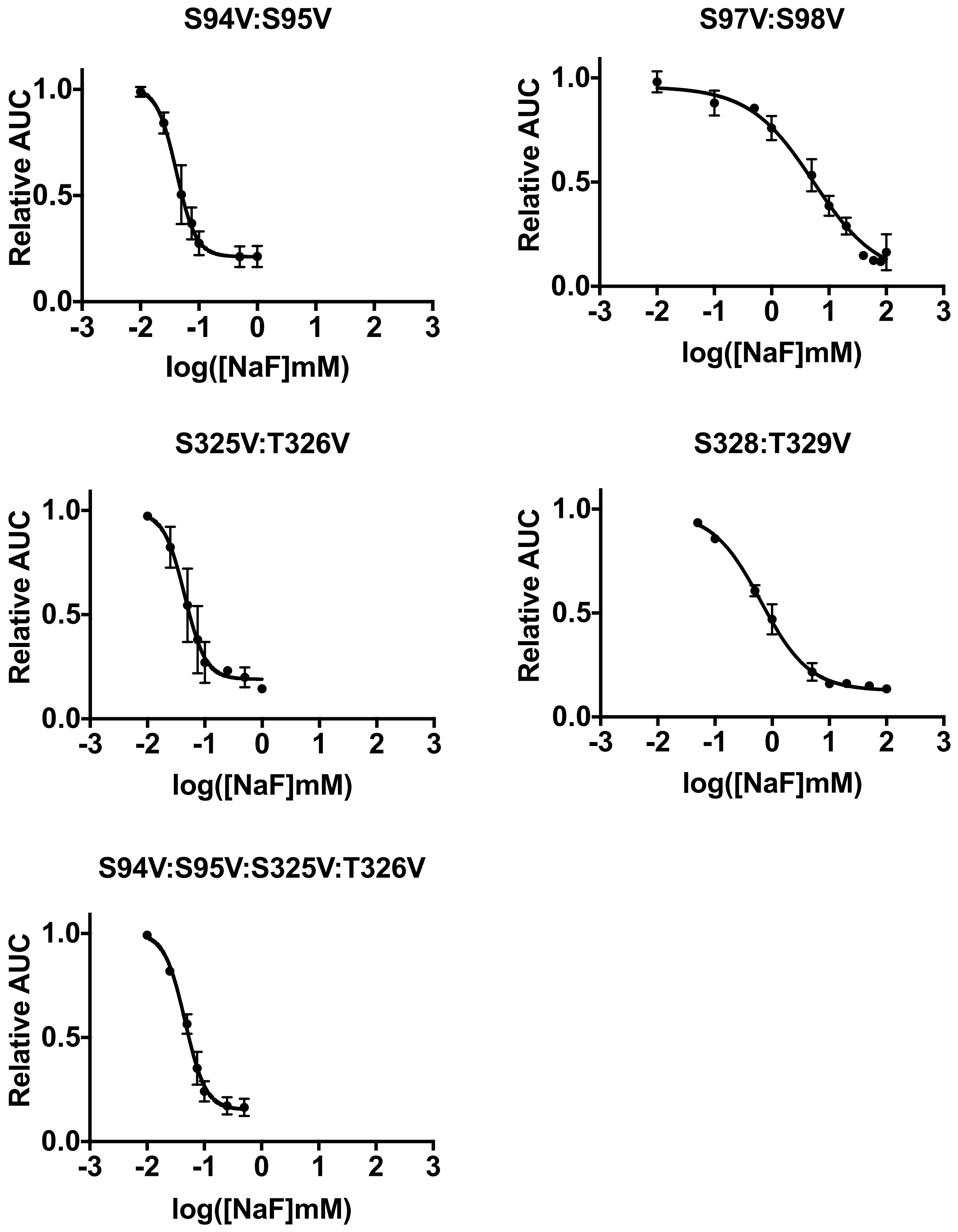

Figure I. Growth curves of yeast expressing FEX proteins with mutations at conserved S/T-rich motifs.

Supplemental References

Li, Sanshu, Kathryn D. Smith, Jared H. Davis, Patricia B. Gordon, Ronald R. Breaker, and Scott A. Strobel. 2013. "Eukaryotic resistance to fluoride toxicity mediated by a widespread family of fluoride export proteins." *Proceedings of the National Academy of Sciences of the United States of America* 110 (47): 19018–19023.

Smith, Kathryn D., Patricia B. Gordon, Alberto Rivetta, Kenneth E. Allen, Tetyana Berbasova, Clifford Slayman and Scott A. Strobel. 2015. "Yeast Fex1p Is a Constitutively Expressed Fluoride Channel with Functional Asymmetry of its Two Homologous Domains." *Journal of Biological Chemistry* 290 (32): 19874–19887.
